# Physiological and immunological changes in the brain associated with lethal eastern equine encephalitis virus in macaques

**DOI:** 10.1371/journal.ppat.1009308

**Published:** 2021-02-03

**Authors:** Joseph R. Albe, Henry Ma, Theron H. Gilliland, Cynthia M. McMillen, Christina L. Gardner, Devin A. Boyles, Emily L. Cottle, Matthew D. Dunn, Jeneveve D. Lundy, Katherine J. O’Malley, Noah Salama, Aaron W. Walters, Ivona Pandrea, Tobias Teichert, William B. Klimstra, Douglas S. Reed, Amy L. Hartman

**Affiliations:** 1 Center for Vaccine Research, School of Medicine, University of Pittsburgh, Pittsburgh, Pennsylvania, United States of America; 2 Department of Infectious Diseases and Microbiology, School of Public Health, University of Pittsburgh, Pittsburgh, Pennsylvania, United States of America; 3 Department of Pathology, School of Medicine, University of Pittsburgh, Pittsburgh, Pennsylvania, United States of America; 4 Department of Psychiatry, University of Pittsburgh School of Medicine, Pittsburgh, Pennsylvania, United States of America; 5 Department of Bioengineering, University of Pittsburgh, Pittsburgh, Pennsylvania, United States of America; 6 Department of Immunology, School of Medicine, University of Pittsburgh, Pittsburgh, Pennsylvania, United States of America; Emory University, UNITED STATES

## Abstract

Aerosol exposure to eastern equine encephalitis virus (EEEV) can trigger a lethal viral encephalitis in cynomolgus macaques which resembles severe human disease. Biomarkers indicative of central nervous system (CNS) infection by the virus and lethal outcome of disease would be useful in evaluating potential medical countermeasures, especially for therapeutic compounds. To meet requirements of the Animal Rule, a better understanding of the pathophysiology of EEEV-mediated disease in cynomolgus macaques is needed. In this study, macaques given a lethal dose of clone-derived EEEV strain V105 developed a fever between 2–3 days post infection (dpi) and succumbed to the disease by 6 dpi. At the peak of the febrile phase, there was a significant increase in the delta electroencephalography (EEG) power band associated with deep sleep as well as a sharp rise in intracranial pressure (ICP). Viremia peaked early after infection and was largely absent by the onset of fever. Granulocytosis and elevated plasma levels of IP-10 were found early after infection. At necropsy, there was a one hundred- to one thousand-fold increase in expression of traumatic brain injury genes (LIF, MMP-9) as well as inflammatory cytokines and chemokines (IFN-γ, IP-10, MCP-1, IL-8, IL-6) in the brain tissues. Phenotypic analysis of leukocytes entering the brain identified cells as primarily lymphoid (T, B, NK cells) with lower levels of infiltrating macrophages and activated microglia. Massive amounts of infectious virus were found in the brains of lethally-infected macaques. While no infectious virus was found in surviving macaques, quantitative PCR did find evidence of viral genomes in the brains of several survivors. These data are consistent with an overwhelming viral infection in the CNS coupled with a tremendous inflammatory response to the infection that may contribute to the disease outcome. Physiological monitoring of EEG and ICP represent novel methods for assessing efficacy of vaccines or therapeutics in the cynomolgus macaque model of EEEV encephalitis.

## Author summary

Eastern equine encephalitis virus (EEEV) is a mosquito-transmitted virus that can cause highly lethal encephalitis in a subset of infected humans. Due to being highly virulent and infectious by aerosol, a need exists to develop vaccines or therapeutics to prevent lethal disease, and FDA approval of such an intervention would likely require use of the FDA’s Animal Rule. To further characterize the cynomolgus macaque model of EEEV after aerosol exposure, we conducted a detailed analysis of the pathological progression of encephalitis in these animals. Physiological condition of the animals was assessed after infection using electroencephalography (EEG) and intracranial pressure (ICP) along with immunological characterization of the inflammatory cells in the brain. We found overwhelming virus infection of the CNS in fatal infections coupled with a strong CNS inflammatory response. Novel findings by EEG and ICP will guide comprehensive studies evaluating the efficacy of medical countermeasures.

## Introduction

Eastern equine encephalitis virus (EEEV; family *Togaviridae*; genus alphavirus) is a mosquito-transmitted RNA virus originally named in the 1800’s for the disease it causes in horses. EEEV causes natural mosquito-borne human infections in North America that peak in late summer months [1]. EEEV encephalitis has a high case fatality rate (CFR), which ranges from 30–70% in human cases [2,3]. In 2019, an unusually large outbreak of 38 human cases of EEEV encephalitis occurred in the northeastern United States, raising fears of further emergence [4]. Serum surveillance suggests, however, that the rates of encephalitis caused by EEEV infection in humans may be low, with roughly 2% of adults and 6% of children infected with EEEV developing encephalitis during the course of natural infection [5].

In humans, disease caused by EEEV is preceded by a limited viral prodrome of nausea, vomiting, fever, general malaise, and/or headache that occurs shortly before or coincident with signs of neurological disease [1,2]. Viremia can occur during this viral prodrome, but is thought to decline before signs of neurological disease manifest [6]. Neurological signs of disease correlate with poor patient outcomes, and fulminant CNS disease can have manifestations such as focal cranial nerve deficits, seizures, or coma. Other neurological issues detectable clinically include generalized decrease in brain electrical activity measured by electroencephalogram (EEG), and brain parenchyma lesions in the basal ganglia, thalamus, and cerebral cortex visible by computed tomography (CT) or magnetic resonance imaging (MRI) [1,2] [7–12]. As with other encephalitic alphaviruses, EEEV is infectious when aerosolized, and there is concern that EEEV could be used as a biological weapon.

Rhesus and cynomolgus macaques were among the first animal species used for modelling EEEV disease. In 1939, Wyckoff & Tesar demonstrated that intranasal inoculation of rhesus macaques with EEEV caused a severe, fatal disease that resembled severe human EEEV cases [13]. Work in macaques was largely absent until 2007, when Reed et al. reported that in cynomolgus macaques, inhalation of a small particle aerosol containing EEEV was fatal at high doses [14]. Macaques developed fever and tachycardia beginning four days after exposure; animals that became febrile succumbed within 36–48 hours of fever onset. Neutrophil counts in the blood, alkaline phosphatase, blood urea nitrogen, and maximum temperature deviation all correlated with outcome. Virus titers in the brain were extraordinarily high, averaging 9 log_10_ pfu/g of tissue.

Further development of the cynomolgus macaque model after aerosol exposure to EEEV is needed for use in pivotal efficacy studies under the FDA’s Animal Rule. Prior studies had been done with cell-culture passaged isolates from mosquitoes, whereas the FDA’s guidance on the Animal Rule requires well-characterized, minimally-passaged human isolates [15]. In addition, while previous studies indicated that fever was the most sensitive indicator of disease, it was also the least specific to neurological disease. Other biomarkers are needed that might be suitable as either a ‘trigger-to-treat’ for therapeutic studies or an indicator of outcome. Finally, to meet other requirements of the Animal Rule, a better understanding of the pathophysiology of EEEV-mediated disease in cynomolgus macaques is needed.

As a mechanism to understand the pathophysiology of EEEV infection and provide a rigorous, reproducible therapeutic evaluation model, we have utilized virus derived from a cDNA clone of the low passage EEEV human isolate, V105, and developed standardized procedures for production of stocks and validation of their potency. Furthermore, we employed radiofrequency telemetry modalities that included continuous EEG and intracranial pressure (ICP) monitoring in addition to body temperature. The study of EEG and ICP adds two dimensions of information relevant to the natural history of inhalational alphavirus infection in the macaque model that have largely remained unexplored. Clinical case reports and case series document various signs of neurological disease in EEEV-infected humans. EEG findings from such reports included variable results from individual patients such as generalized decreases in EEG and epileptiform discharges [11,12]. Increased ICP has been reported in human patients with viral encephalitis including EEEV [7].

In addition to radiotelemetry monitoring, we performed a comprehensive analysis of the pathogenesis of EEEV in cynomolgus macaques, including longitudinal and endpoint immune and cytokine responses and inflammatory cell infiltration into the CNS. A careful evaluation of immune mediators and traumatic brain injury markers in blood has not been previously reported for cynomolgus macaques infected with EEEV and warranted consideration as potential biomarkers. The results presented here provide granular insight into the pathogenic progression of EEEV encephalitis and will guide future therapeutic and vaccine trials using cynomolgus macaques.

## Results

### Outcome and severity of fever after aerosol infection of macaques with V105 is dose-dependent

**Fig 1:**
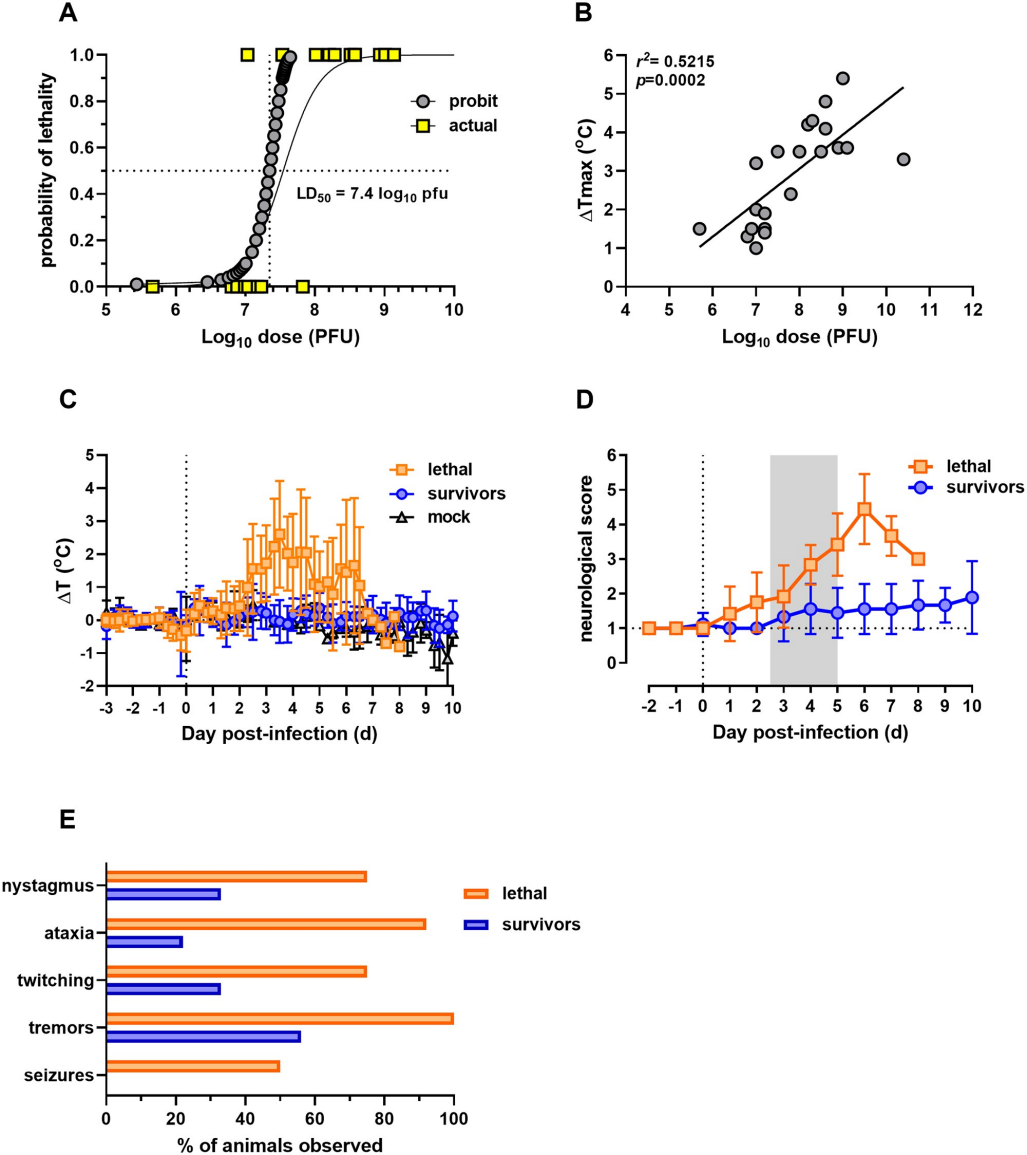
Outcome of aerosolized EEEV V105 infection of cynomolgus macaques. (A) Probit analysis of dose and survival for calculation of approximate LD_50_. (B) Exposure dose correlates with fever severity. Maximum temperature change was compared to inhaled exposure dose. (C) Average 6-hour temperature change for each group. (D) Daily neurological scores during EEEV infection. Gray shaded area indicates average febrile period. (E) Frequency of neurological observations (% of animals in which the listed parameter was observed). For all panels, n = 12 for lethal infections and n = 9 for sublethal infections.

For this study, a total of 21 macaques were exposed to small particle aerosols containing clone-derived EEEV strain V105. Inhaled doses ranged from 5.7 to 10.4 log_10_ PFU (Table 1). Of the 21 animals, 9 survived infection while 12 succumbed to disease within 5–8 days (mean time to death of 6 days; Table 1). Lethally-infected macaques received a significantly higher average inhaled dose than the survivors (range of 7.0–10.4 compared to 5.7–7.8 log_10_ PFU, respectively) with no differences in inhaled dose between male and female groups. Probit analysis of inhaled dose and survival outcome yielded an LD_50_ of 7.4 log_10_ pfu (Fig 1A). In lethally-infected macaques, febrile responses were detected between 2–3 days post-infection (dpi) which peaked on 4–5 dpi and then declined (Fig 1C). Both mock-infected and surviving EEEV-infected macaques had minimal temperature elevations from predicted (mean for mock and survivors T_max_ of 1.5°C; Table 1). In contrast, lethally EEEV-infected macaques had a T_max_ between 3.2–5.4°C elevation (Table 1). T_max_ values strongly correlated with the inhaled dose (Fig 1B).

**Table 1:** Cynomolgus macaque cohort parameters and fever response.

Group	Animal ID	Sex	Age (yr)	Weight	Dose (log10)	TTD	Onset	TMax	Duration	Fever-hours	Ave Elev
**Survivors**	M162-16	M	7.0	8	5.7		0.0	1.81	41.5	30.5	0.7
	M113-18	F	7.5	6.3	6.8		0.0	2.35	165.3	147.6	0.9
	M114-18	F	5.1	3.1	6.9		0.0	2.50	173.0	169.5	1.0
	M118-18	M	8.8	7.5	7.0		0.5	2.62	348.8	377.4	1.1
	M160-16	M	7.0	6.6	7.0		0.0	0.69	14.3	6.5	0.5
	M110-18	F	6.0	3.5	7.2		2.5	1.91	89.0	79.0	0.9
	M123-16	F	6.0	4.8	7.2		0.0	1.63	40.8	68.1	1.7
	M108-18	F	6.0	3.4	7.2		5.0	1.59	22.0	26.5	1.2
	M4-19	M	2.0	2.7	7.8		0.0	2.40	78.3	93.2	1.2
**Lethal**	M120-16	F	5.0	3.8	7.0	5	1.5	3.23	67.0	123.1	1.8
	M163-16	M	7.0	6.6	7.5	6	2.8	4.40	65.0	154.9	2.4
	M1-19	F	2.0	2.3	8.0	6	2.5	3.52	68.8	150.0	2.2
	M161-16	M	7.0	5.6	8.2	6	3.3	4.21	70.8	144.2	2.0
	M2-19	F	2.0	2.3	8.3	6	1.3	4.30	89.3	200.2	2.2
	M118-16	M	5.0	7.4	8.5	7	5.0	3.82	63.3	120.9	1.9
	M57-17	M	5.0	6	8.6	5	2.3	5.33	81.3	222.4	2.7
	M3-19	M	2.0	2.7	8.6	5	2.5	4.11	63.5	142.3	2.2
	M119-16	M	6.0	7.2	8.9	5	3.0	4.53	51.8	111.9	2.2
	M58-17	M	5.0	5.5	9.0	6	2.3	5.46	107.0	254.8	2.4
	M160-17	M	6.0	6.6	9.1	6	3.5	3.61	88.8	133.9	1.5
	M163-17	M	5.0	8.1	10.4	8	5.3	4.29	78.0	99.4	1.3
**Mock**	M115-16	M	6.0	4.62	0		0.0	1.65	53.3	39.2	0.7
	M116-16	M	5.0	5.2	0		0.0	1.36	25.0	19.4	0.8

Weight is in kg.

Dose (log10) is in pfu.

TTD = time to death in days.

Onset = day of fever onset (>4 hr sustained fever).

Tmax = maximum deviation from predicted.

Duration = fever duration in hours.

Fever-hours = sum of significant temperature elevations.

Ave Elev = average elevation during fever response.

NT = not tested.

Infected NHPs were monitored carefully for clinical signs of disease and scored at least twice daily. Most infected macaques, regardless of final outcome, had general non-specific signs of illness such as decreased appetite and activity. Illness in surviving animals was significantly less severe than the lethal infections, as depicted in the neurological scores (Fig 1D). Animals were observed by study staff at least twice daily, and neurological scores increased beginning with fever onset and peaked on day 6 post-exposure. Nystagmus, ataxia and twitching were noted in both lethally-infected and surviving macaques but were more prominent and occurred more frequently in lethal infections. Tremors were observed in all infected animals; however, tremors in surviving animals were mild compared to those observed in lethally-infected macaques (Fig 1E). Tonic-clonic seizures were observed directly or by video recordings in 50% of the lethal infections and were considered a near end-stage event. No seizures were noted in macaques that survived EEEV infection.

### Disruption in EEG and ICP patterns in lethally-infected macaques

A subset of animals were implanted with telemetry devices to measure EEG, including 2 uninfected controls. The EEG leads were placed at approximate F4 and O1 positions on the 10–20 EEG map such that the two leads spanned the brain from front to back [16]. EEG measures global fluctuations in ionic electrical currents stemming from neuronal activity in the brain, and analysis of different frequency components of the waveform can aid the identification of pathological neurologic states as may be encountered in acute viral encephalitis [11,12,17,18]. Raw EEG trace data were resolved into delta (δ), theta (Ɵ), alpha (α), and beta (β) wave bands. The significance of slow-wave frequencies such as those covered by the delta and theta ranges lies in their association with non-rapid eye movement sleep (NREM), during which delta wave signal peaks [19–21]. Slow-wave frequencies are associated with restful states including decreased conscious thought and focus. In contrast, higher frequency waves (alpha and beta wave bands) are associated with voluntary movement, feedback, and muscular coordination [22–24].

**Fig 2:**
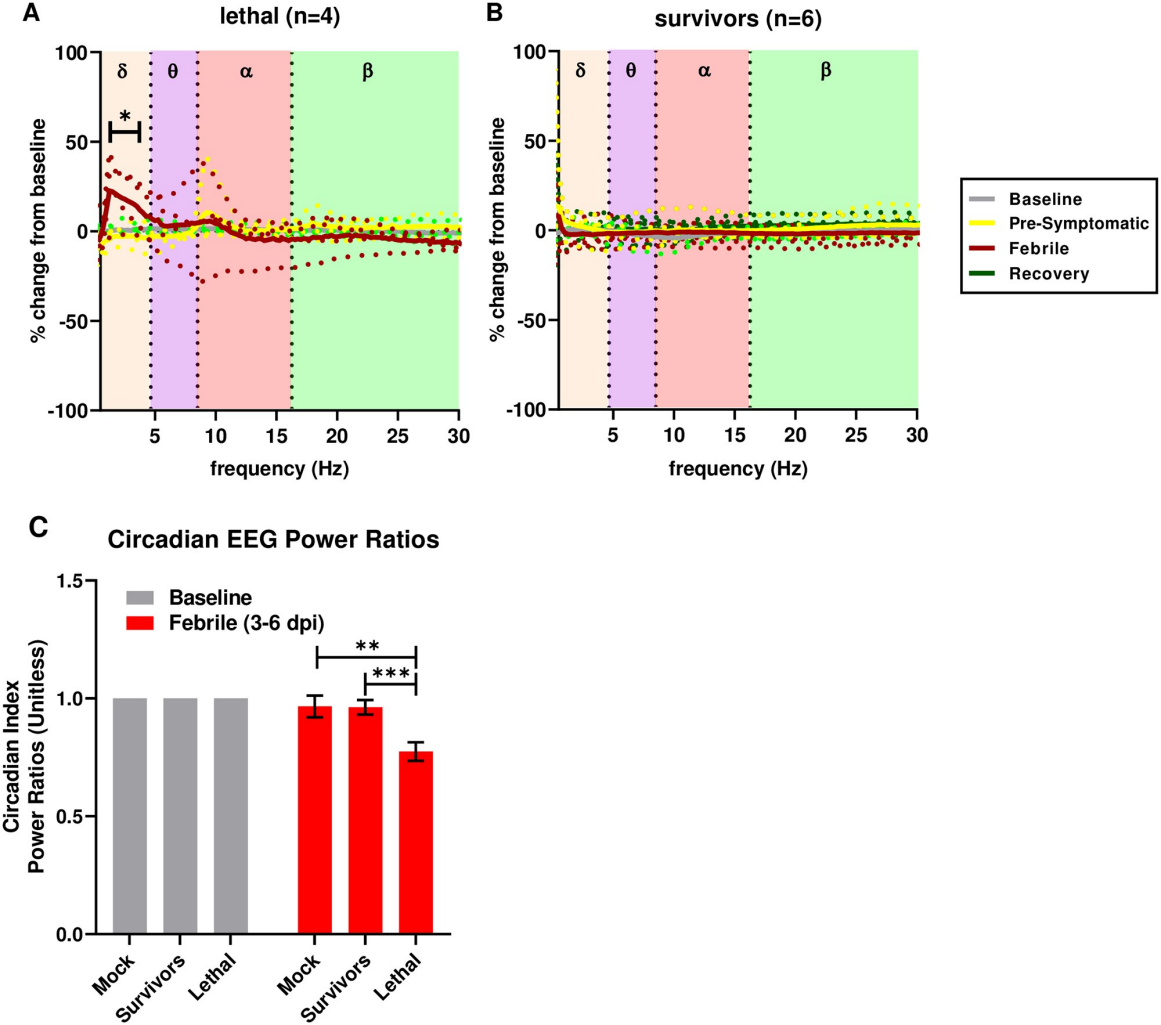
Febrile periods are associated with increased delta band activity. Power spectral density plotted as percent change from baseline in cohorts of EEEV-infected macaques in (A) lethal (n = 4) and (B) survivor (n = 6) groups. Each phase of infection is indicted by a different color. Solid lines represent group averages with individual animals in dotted lines. Statistically significant increases in delta activity occurred in the lethal EEEV group during the febrile period (red line in B) (p <0.01). (C) Power ratios of circadian indices compared between febrile and baseline periods. Repeated measures ANOVA demonstrate decreased periodogram amplitudes in macaques with lethal EEEV compared to survivors (p = 0.0002) and mock-infected (p = 0.0039) macaques, suggestive of suppression of circadian variation in the macaques with lethal EEEV.

Power spectral density plots of EEG patterns were generated for the various stages of disease (baseline, pre-symptomatic, febrile, recovery). A significant increase in the delta wave power band (22.4%) was observed during the febrile phase for lethally-infected macaques (Fig 2A). While other trends appear to exist, such as increases or decreases in alpha or theta activity for individual macaques, these effects did not elicit statistical significance at the group level. In contrast, no significant changes were noted in any surviving macaques at any point after exposure (Fig 2B). Circadian index, the ratio of the delta power band to the beta power band, was also evaluated. Circadian indices of lethally-infected animals were significantly disrupted during the febrile period while no changes were noted in mock-infected macaques or survivors (Fig 2C).

**Fig 3:**
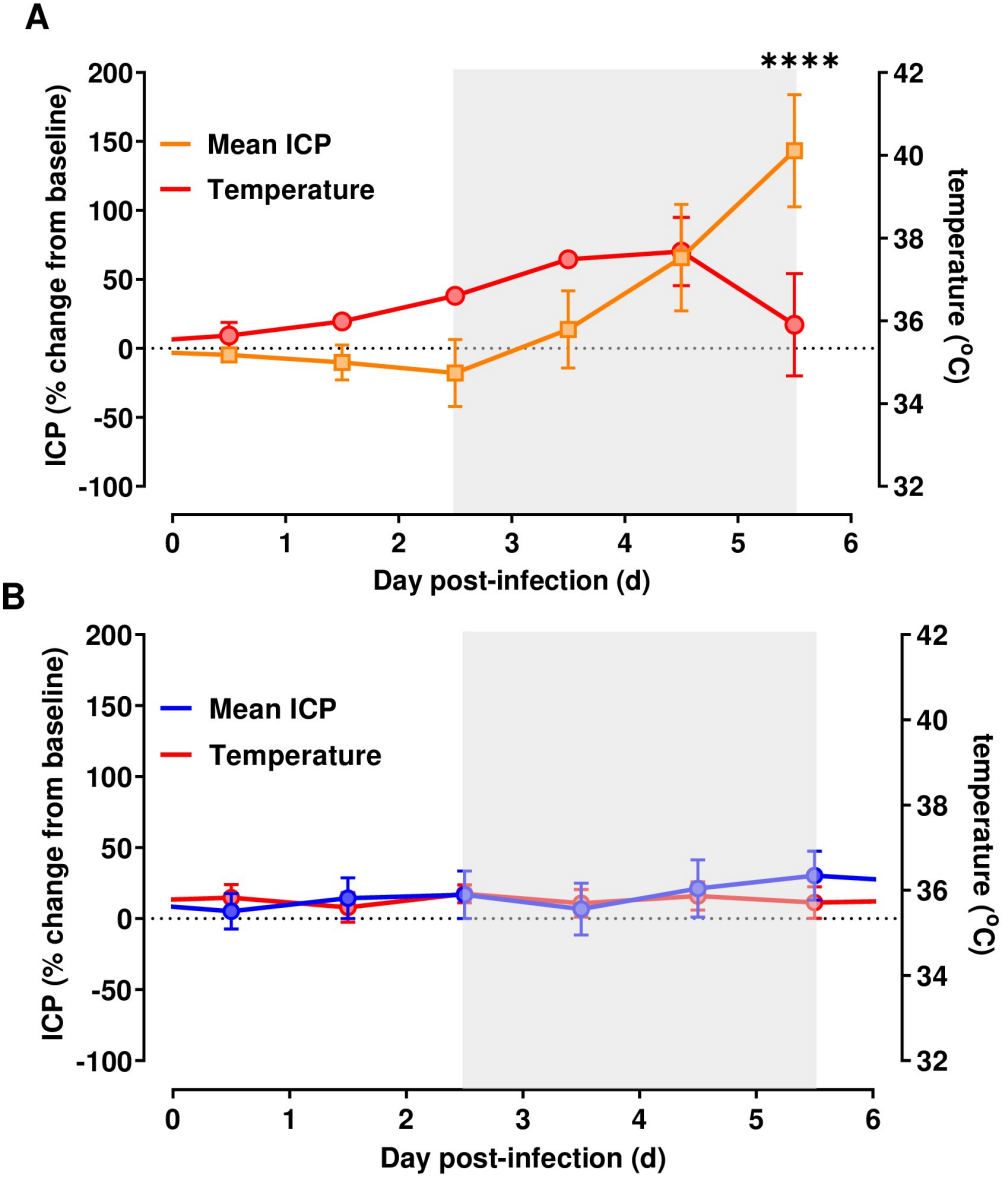
Intracranial pressure increases during lethal EEEV infection. ICP (% change from baseline +/- SEM) in EEEV-infected macaques during (A) lethal disease (n = 4) and (B) survivors (n = 6), each superimposed upon aggregate temperature data (red line) for the same cohort. Gray box indicates febrile period in lethally-infected cohort. Repeated measures ANOVA with post-hoc pairwise comparisons found significant increases in ICP compared to mock-infected macaques on day 6 post infection, denoted by the asterisks (p<0.0001).

ICP was also evaluated along with changes in EEG and body temperature. Beginning on 4 dpi, mean daily ICP begins to rise in lethally-infected macaques (Fig 3A) and by 6 dpi, it was 143% above baseline. In contrast, the percentage change in ICP stayed below 50% on any given day post infection for the group of surviving EEEV-infected macaques in the six-day period typical of survival for lethal macaque infections and never differed significantly from baseline (Fig 3B).

### Hematological findings

**Fig 4:**
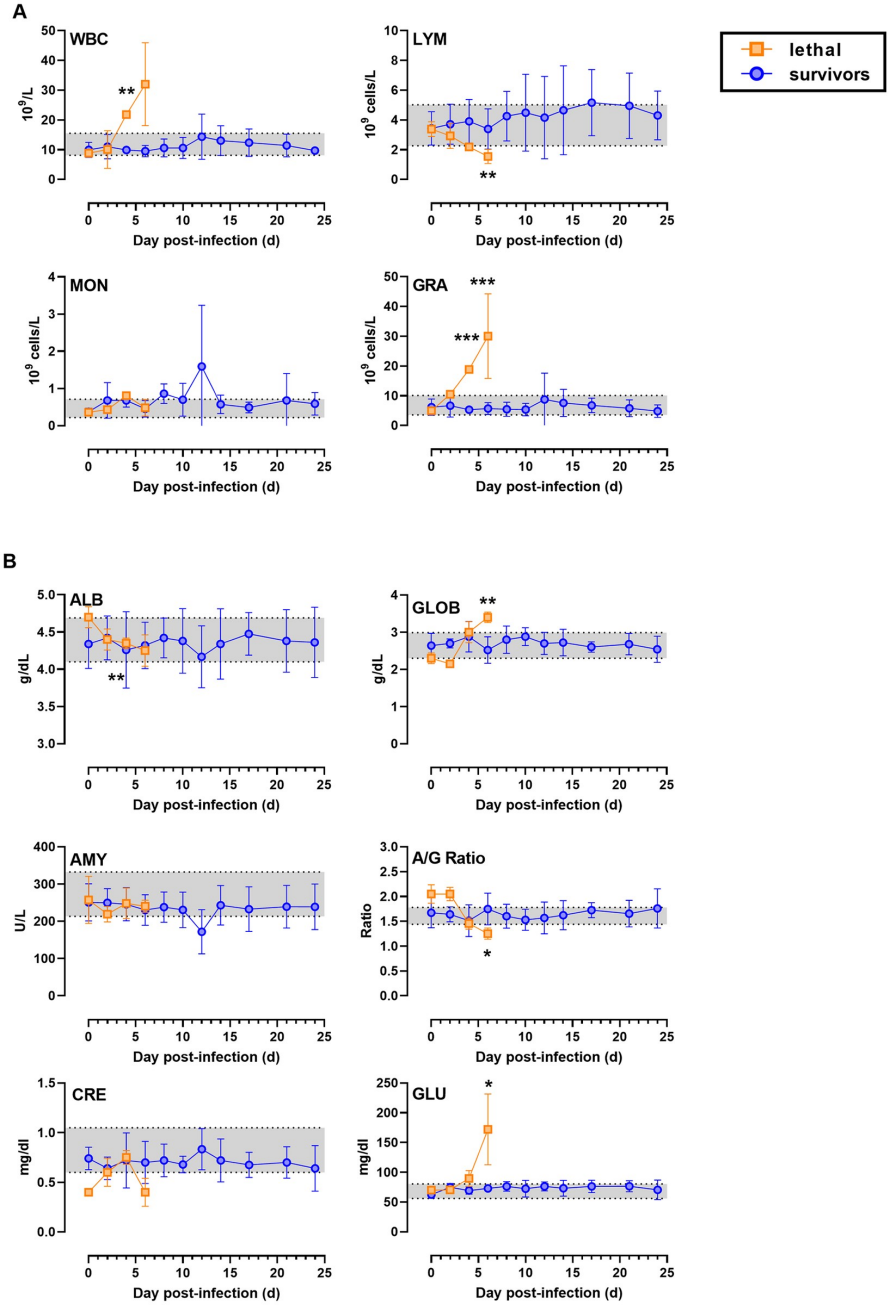
Lethal EEEV infection is associated with granulocytosis and lymphopenia. A subgroup of 9 animals (3 lethal; 6 sublethal) were sampled longitudinally every 2 days. Grouped data are shown for (A) CBC and (B) clinical chemistries. WBC, white blood cells; LYM, lymphocytes; MON, monocytes; GRA, granulocytes; ALB, albumin; GLOB, globulin; AMY, amylase; A/G ratio, albumin:globulin ratio; CRE, creatinine; GLU, glucose. Statistical significance was determined by 2-way ANOVA with multiple comparisons. Time points were compared to 0 dpi for each group and significance is indicated by the number of the asterisks above each time point.

**Fig 5:**
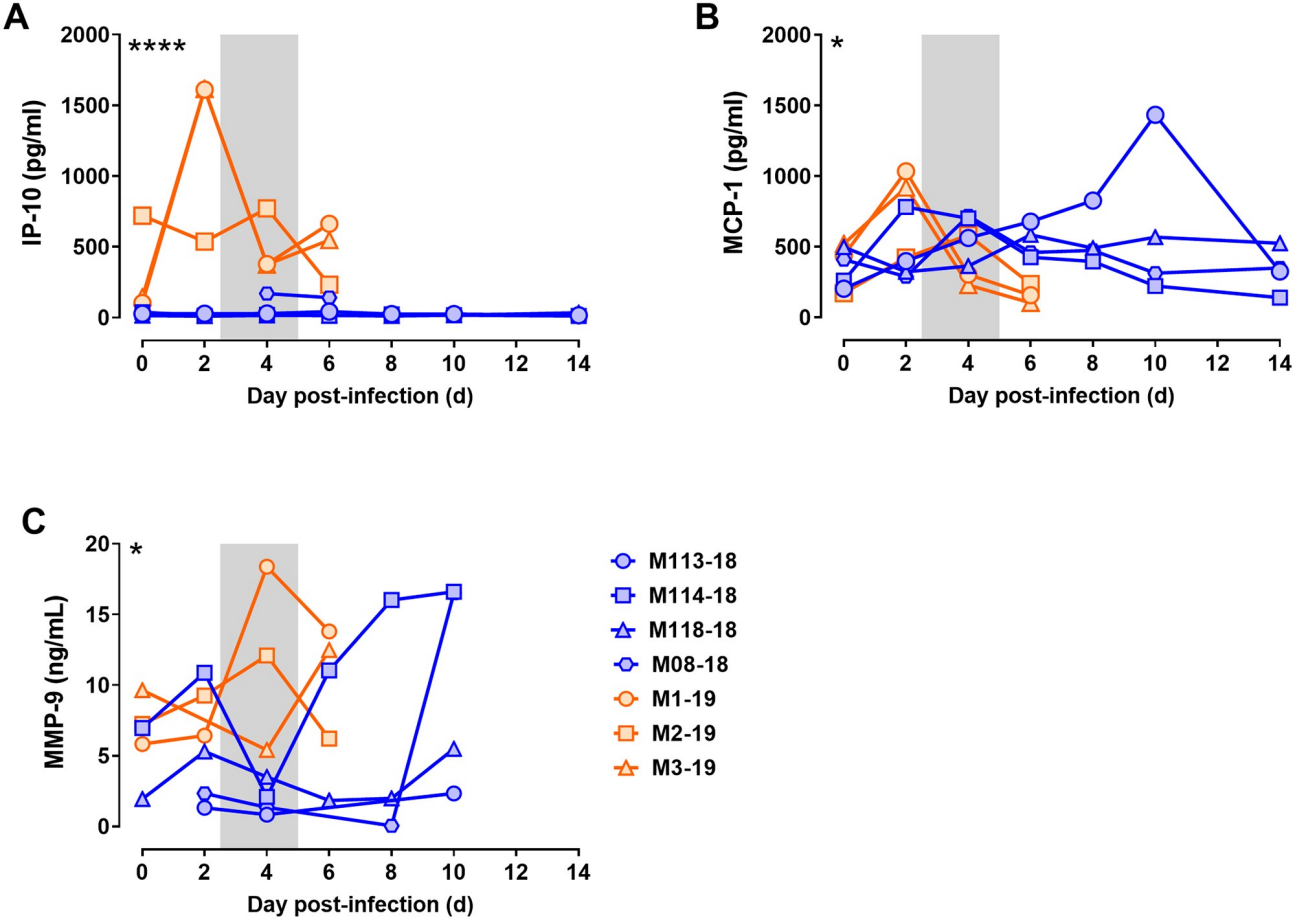
Longitudinal plasma cytokines in EEEV-infected macaques. Cytokines were measured by Legendplex assay or ELISA. (A) IP-10. (B) MCP-1. (C) MMP-9. Shaded area represents febrile period. Orange = lethal infections; blue = survivors. For statistical comparisons, two-way ANOVA was used to compare survivors and lethal infections from 0–6 dpi and significance is indicated by the asterisks in the upper left corner of each graph.

A subset of 9 macaques (3 lethal; 6 sublethal) had blood drawn and swabs taken longitudinally every 2 days to assess clinical laboratory changes associated with severe disease and viremia. A significant elevation in white blood cells (WBC) was seen on 4 dpi in lethally-infected macaques, which was comprised entirely of granulocytes (Fig 4A). Lymphocyte counts, in contrast, declined significantly by day 6 in lethally-infected macaques. No significant changes in total WBC or any subsets were noted in surviving animals. Clinical chemistries noted significant increases in globulin and glucose and decreases in albumin and the albumin:globulin ratio (A/G) during lethal infection (Fig 4B). Significant changes in any other clinical chemistries were not noted. Analysis of cytokines and chemokine levels in the blood of EEEV-infected macaques found elevated levels of IP-10 at 2, 4, and 6 dpi in three lethally-infected macaques but not in survivors (Fig 5A). No significant differences were seen in MCP-1 between survivors and lethally-infected macaques (Fig 5B). MMP-9 was elevated in two of three lethally-infected macaques at 4 dpi and late in two of four survivors (Fig 5C). No other differences were seen in other cytokines and chemokines evaluated in the blood.

### No differences in viremia between survivors and lethal infections

**Fig 6:**
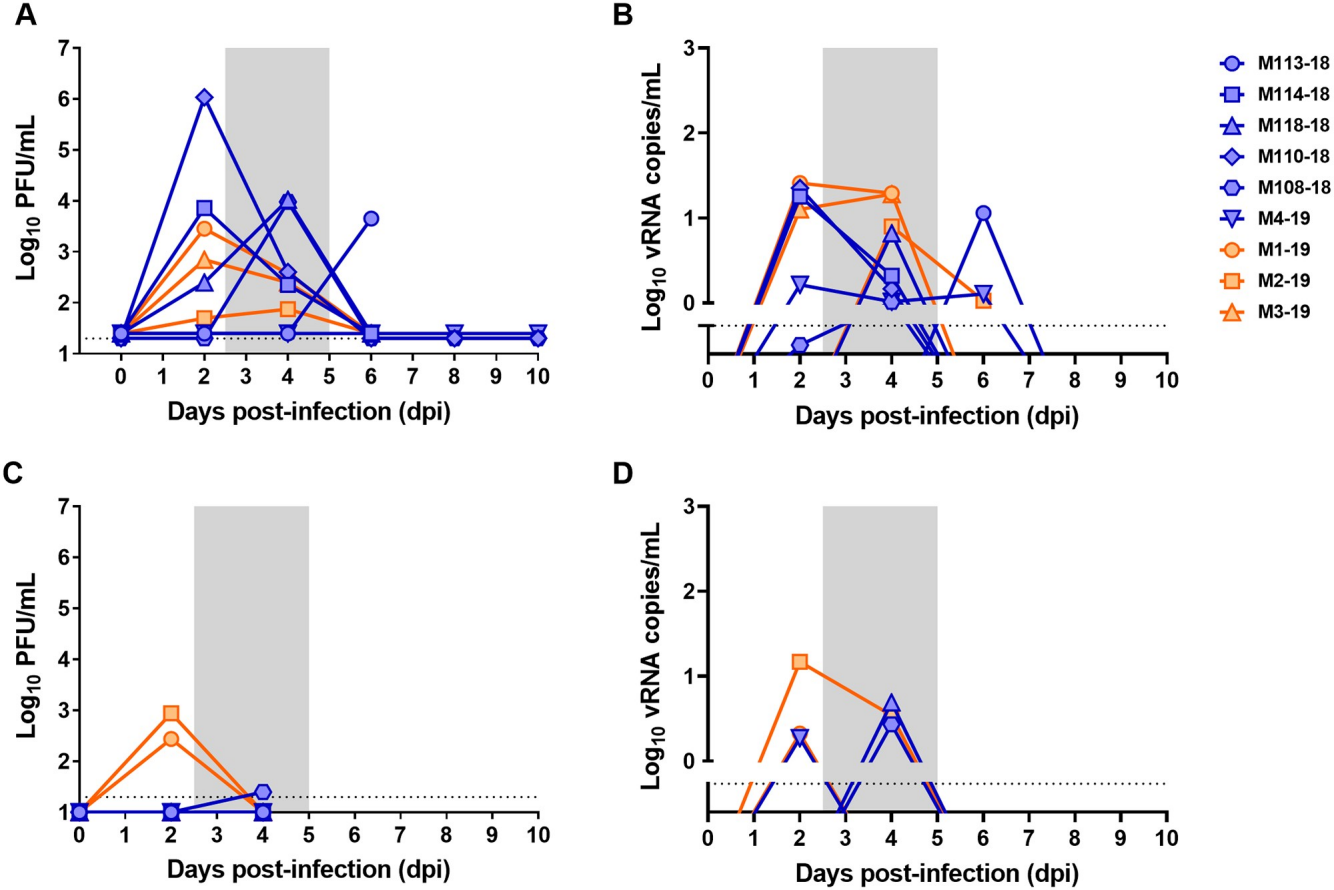
Viremia and nasal shedding of EEEV V105 after infection. Blood and nasal swabs were taken every 2 days from the 9 animals that were sampled longitudinally. (A) and (B) are plasma samples; (C) and (D) are nasal swabs. Infectious virus was measured by plaque assay in (A) and (C). Viral RNA was measured by q-RT-PCR in identical samples in (B) and (D). Orange represents lethally-infected animals (n = 3); blue represents surviving animals (n = 6). There was no difference in timing or magnitude between the two groups. Shaded area represents febrile period.

Despite significant differences in febrile responses and neurological scores between lethal infections and survivors, transient viremia was detected in plasma by both plaque assay and qRT-PCR from 2–4 days after exposure, regardless of survivor status (Fig 6A and 6B). No significant differences were noted in the viremia levels between survivors and lethal infections. Low levels of infectious virus and vRNA were also detectable in nasal swabs of both lethally-infected and surviving macaques (Fig 6C and 6D).

### High viral load in post-mortem tissues from lethal infections

**Fig 7:**
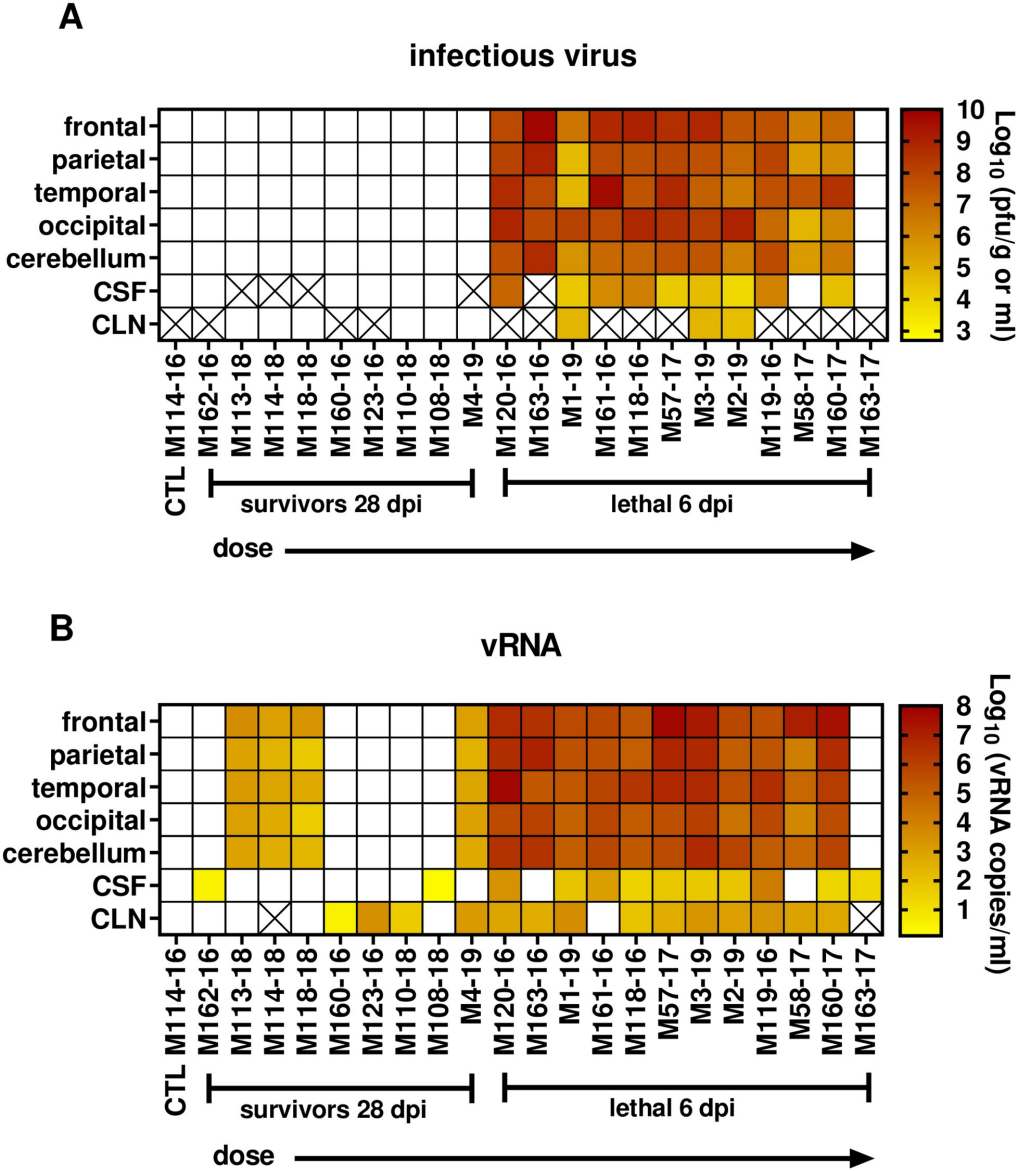
Virus levels in the brain of EEEV-infected NHP. Virus levels were measured by (A) plaque assay and (B) q-RT-PCR in the indicated tissues at necropsy. Lethal infections were necropsied at approximately 6 dpi; survivors at 28 dpi. Heatmaps represents log-transformed titers after background subtraction. X through the square indicates sample was not available for testing.

At the time of euthanasia (average of 6 dpi for lethal infections; 28 dpi for all survivors), a full necropsy was conducted including collection of cerebrospinal fluid (CSF) and dissection of the brain. Lethally-infected NHP had very high levels of both infectious virus and vRNA throughout tested brain regions; virus was also found in the cerebrospinal fluid (CSF) and cervical lymph nodes (CLN) (Fig 7). Virus was evenly distributed throughout the lobes of the cortex and the cerebellum, indicating that virus infection was not limited to a specific brain region at end-stage disease. Virus levels in the CSF were 3–5 logs lower than the brain tissue itself. In surviving animals, no infectious virus was detected at the time of necropsy (28 dpi), although some residual viral RNA was detectable in the brain and/or CLN of 4/9 survivors (Fig 7B), and notably these survivors demonstrated the highest number of fever-hours of all of the surviving animals (Table 1).

### Inflammatory cytokines and TBI markers in CNS tissues from lethal infections

**Fig 8:**
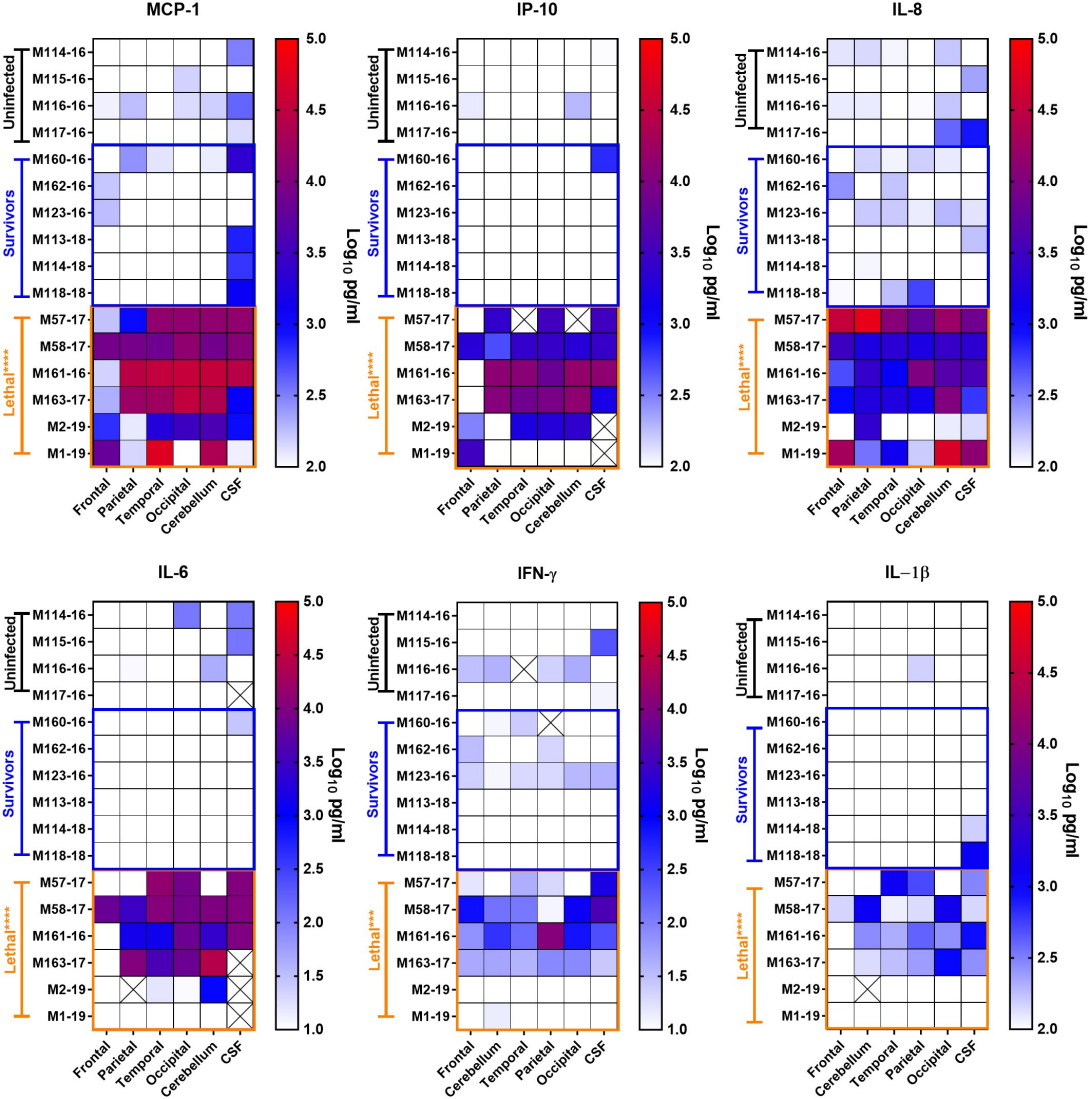
Cytokine and chemokine levels in the brain of EEEV-infected NHP. Multiplex analysis was used to measure the indicated cytokines in homogenized tissue samples from necropsy. Lethally infected animals were necropsied at approximately 6 dpi; surviving animals at 28 dpi. For all cytokines shown, values in lethally infected animals were significantly elevated over the uninfected and survivor animals (indicated by orange asterisks). X through the square indicates sample was not available for testing.

Significantly elevated levels of several inflammatory cytokines and chemokines (MCP-1, IP-10, IL-8, IL-6, IFN-γ, and IL-1β) were found in the brains of the lethally-infected macaques (Fig 8). In particular, MCP-1, IP-10, IL-8 and IL-6 were elevated 3–5 logs across all regions of the brain examined above both mock-infected and surviving macaques, although there was some variation between regions in individual macaques. IFN-γ and IL-1β were significantly elevated but to a lesser degree than the other 4 analytes, and in two lethally-infected macaques were not elevated at all. Despite the fact that the CSF had 3–5 logs lower levels of virus compared to brain tissue, cytokine and chemokine levels in CSF were similar to the brain tissue.

**Fig 9:**
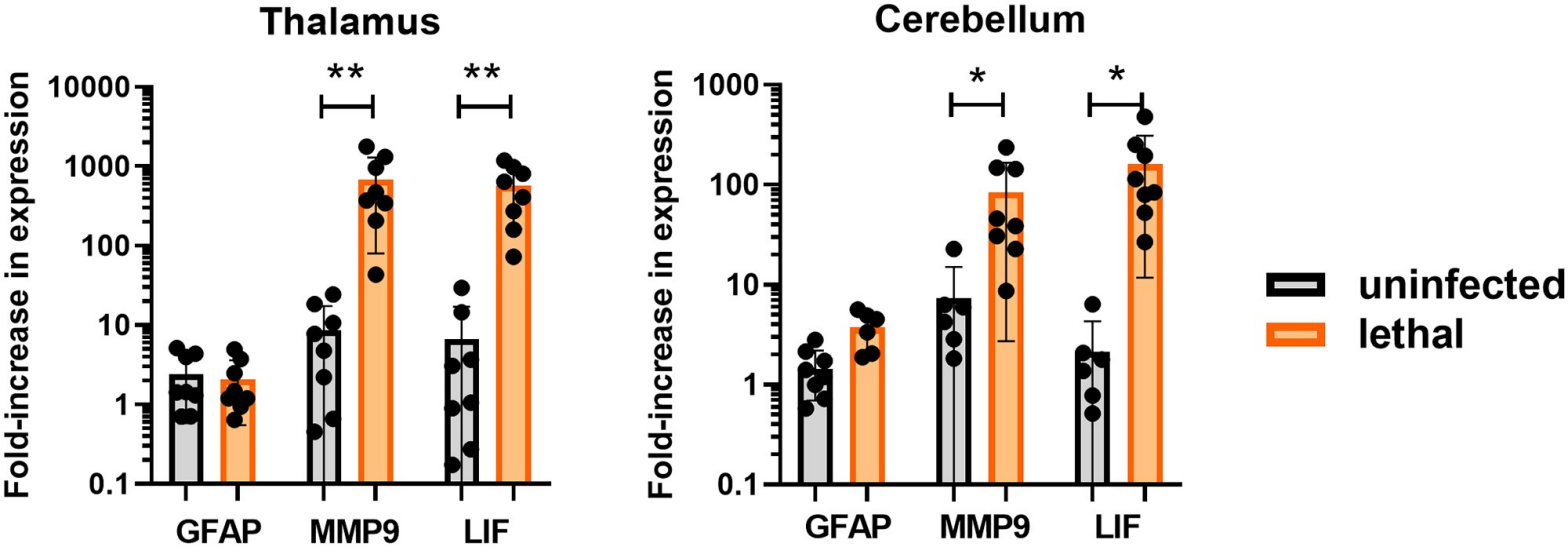
Expression of markers of traumatic brain injury in the CNS of EEEV-infected macaques. q-RT-PCR was used to measure the relative fold increase in expression of each indicated marker. GFAP, glial fibrillary acidic protein; MMP-9, matrix metalloproteinase 9; LIF, leukemia inhibitory factor. Y-axis represents the geometric means of fold-upregulation. Error bars represent standard error of the mean. Asterisks indicate significance as measured by t-test.

Soluble mediators released due to traumatic brain injury (TBI) represent potential biomarkers of viral encephalitis [25–28]. RNA isolated from the thalamus and cerebellum of lethally-infected EEEV and mock-infected macaques was assessed by q-RT-PCR for expression of three potential markers of TBI: glial fibrillary acidic protein (GFAP), leukemia inhibitory factor (LIF), and MMP-9 (Fig 9). Expression of MMP-9 and LIF genes were increased 10 to100-fold in lethally-infected EEEV macaques in both the thalamus and cerebellum compared to expression in mock-infected macaques. GFAP, in contrast, was not significantly elevated in lethally-infected EEEV macaques.

### Lymphocytes infiltrate the brain during both lethal and non-lethal EEEV infection

**Fig 10:**
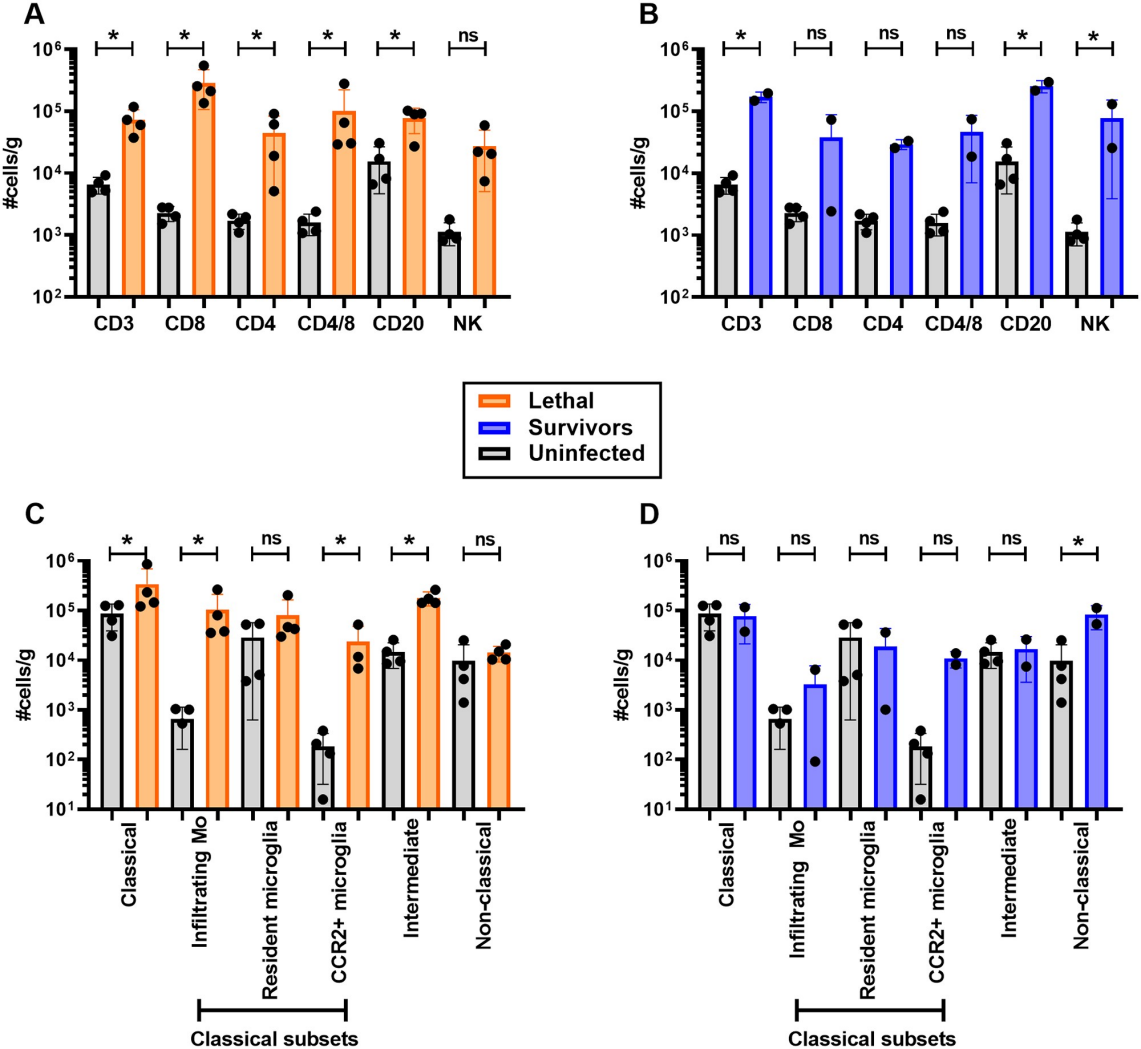
Leukocyte infiltration into the brain of EEEV-infected macaques. Leukocytes were isolated from brain tissue and phenotyped using flow cytometry (gating strategy shown in S1 Fig). (A,B) Lymphoid and (C,D) myeloid lineage cells from uninfected (gray; n = 4), lethal infections (orange; n = 4; 6 dpi), and survivors (blue; n = 2; 28 dpi). For each animal, cells were isolated from 5 brain regions (frontal, temporal, parietal, occipital, cerebellum) and each circle represents the average #cells/gram for each animal for all 5 regions. Bars indicate group averages +/- SD. Statistically significant differences were determined using 2-way ANOVA with multiple comparisons. Significance between uninfected and infected animals is indicated by the asterisk above each group.

Leukocytes isolated from digested brain tissues (frontal, temporal, occipital, parietal, cerebellum) were phenotyped by flow cytometry from uninfected (n = 4), lethally-infected (n = 4), and surviving (n = 2) macaques (S1 Fig). For lymphoid lineage cells including CD3+, CD8+, CD4+, CD4+8+, and CD20+, total cell numbers were significantly elevated in lethally-infected animals at 6 dpi compared to uninfected controls (Fig 10A). The recruitment of lymphocytes and NK cells into the brain corresponds with increased expression of IP-10 within the brain (Fig 8). Interestingly, elevated lymphoid lineage cell numbers remained in the brains of surviving animals at 28 dpi (Fig 10B).

### Infiltration of myeloid cells and activation of microglia in the brain during lethal infections

For myeloid lineage cells, we were able to distinguish monocyte subsets based on expression of CD14 and CD16 (S1 Fig). Classical (CD14+CD16-) and intermediate (CD14+CD16+) monocytes were significantly elevated in lethal brains but not survivors (Fig 10C and 10D). We further analyzed the classical monocytes to distinguish resident microglia based on expression of the Iba-1 microglia marker (CD14+CD16-CD11b+HLA-DR+Iba-1+CCR2-) from infiltrating peripheral macrophages that express the brain-homing CCR2 marker (CD14+CD16-CD11b+HLA-DR+Iba-1-CCR2+) and a hybrid microglia-like macrophage that expresses both Iba-1 and CCR2 (CD14+CD16-CD11b+HLA-DR+Iba-1+CCR2+). The ligand for CCR2 is MCP-1, which was highly elevated in brain and CSF tissue of lethally-infected animals (Fig 8). While the number of resident microglia did not change after infection with EEEV, the infiltrating macrophages and the microglia-like macrophages were both significantly elevated in the lethal EEEV animals, reflecting the higher expression of MCP-1 and recruitment of macrophages to the CNS.

### Severe pathological lesions associated with lethal EEEV encephalitis

**Fig 11:**
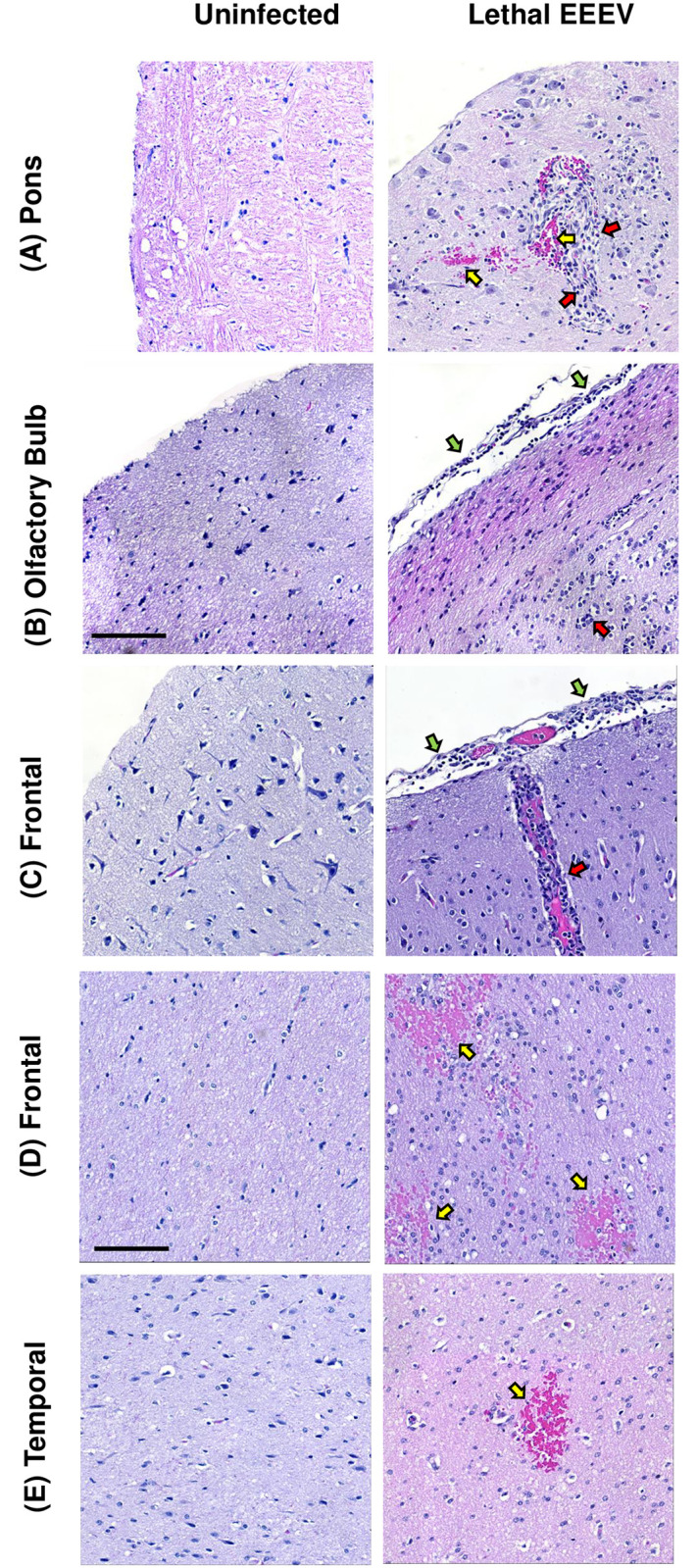
Examples of pathological changes in the brain during lethal EEEV infection. Scale bar is 100 uM. Yellow arrows indicate hemorrhage (red blood cells staining pink); red arrows indicating leukocyte inflammation (small cells that stain dark purple); green arrows indicate meningitis (leukocytes on the outer edge of the meninges). (A) Pons from M118-16 at 7 dpi showing hemorrhage and leukocytic infiltration. (B) Olfactory bulb from M58-17 at 6 dpi showing leukocytic infiltration and meningitis. (C) and (D) Frontal lobe from M160-17 at 6 dpi illustrating leukocytic infiltration, meningitis and hemorrhage. (E) Temporal lobe from M119-16 at 6 dpi showing an example of hemorrhage within the parenchyma.

**Fig 12:**
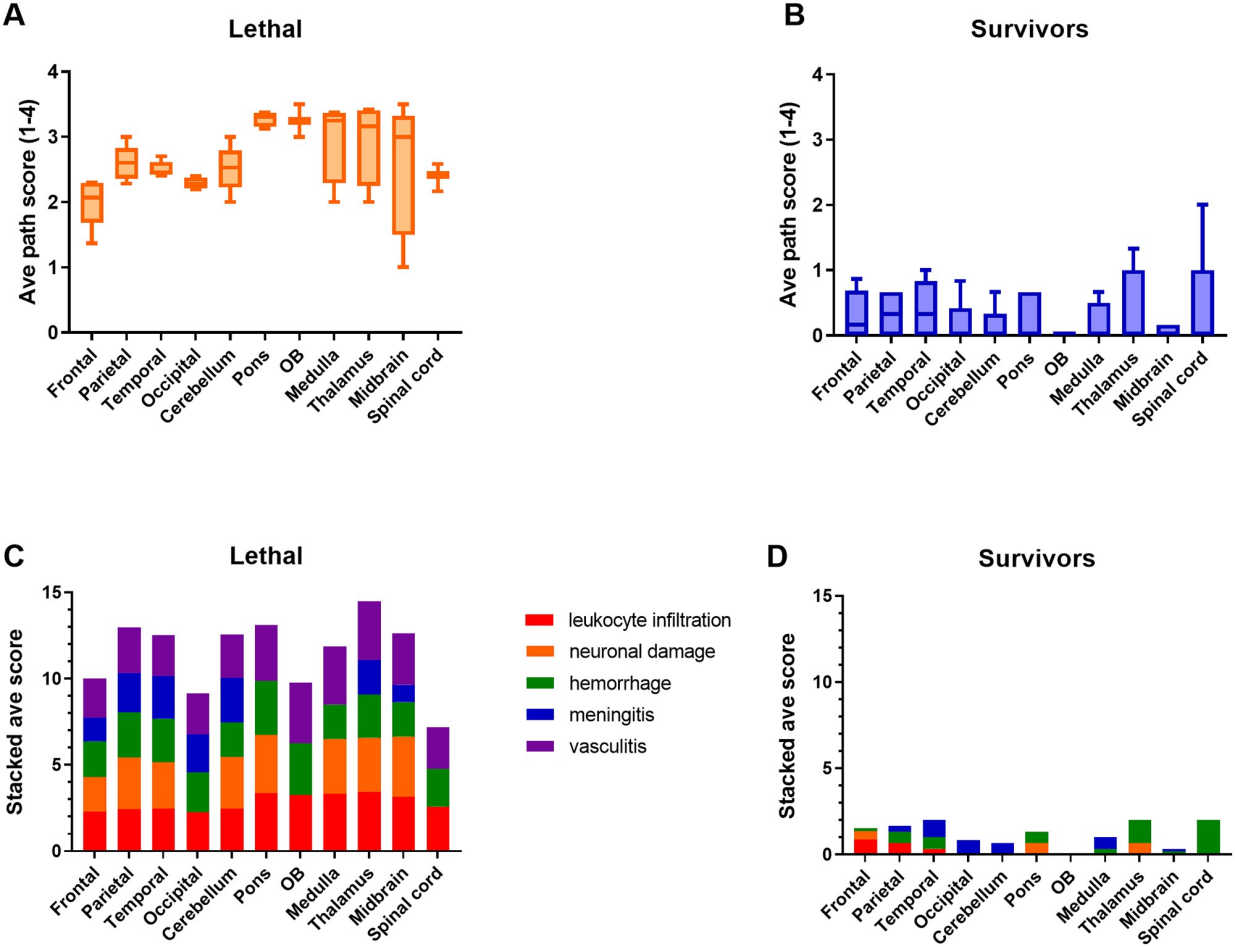
Pathological scoring of brain tissue. Formalin-fixed tissue sections were stained with H&E and scored 0–4 based on severity of lesions. Tissue sections from (A) lethally infected animals were obtained at the time of euthanasia (6–8 dpi; n = 9). Samples from (B) sublethally infected animals were obtained at necropsy (>26 dpi; n = 3). (C) and (D) show the average cumulative stacked score of each lesion type per region.

Virus-induced pathology was confirmed by the microscopic analysis of CNS tissues collected at necropsy. The lethal cases had severe lesions disseminated throughout the brain regions. They included inflammation, necrosis, and hemorrhage (Fig 11). H&E stained tissue sections were scored 0–4 based on severity of lesions (Fig 12). In all regions of the brain examined, lethally-infected macaques had elevated scores compared to survivors. Based on averaged scores across 9 lethally-infected macaques, the most severely affected regions were the pons, olfactory bulb, medulla, thalamus and mid-brain, and the frontal lobe was the least affected. The most frequently noted lesions were: leukocyte infiltration (in the meninges [meningitis], in the brain parenchyma [encephalitis] and in and around blood vessels [vasculitis]), neuronal damage, and hemorrhage. These lesions were relatively uniformly distributed throughout the brain regions (Fig 12C).

## Discussion

The primary objectives of this study were to advance the development of the cynomolgus macaque model after aerosol exposure to EEEV under controlled and reproducible conditions and to identify potential biomarkers for EEEV CNS disease and clinical outcome. A secondary objective was to evaluate V105, an infectious clone of an EEEV isolate from a fatal human case, to determine if it was suitable for use as the challenge material in pivotal studies to evaluate medical countermeasures against EEEV. Prior studies with EEEV in macaques have primarily used mosquito isolates. However, the FDA’s Animal Rule for pivotal efficacy studies to support licensure of drugs and biologics requires that a low-passage human isolate be used rather than an animal or insect isolate [15]. We demonstrate here that aerosol exposure of cynomolgus macaques to high doses of V105 resulted in a significant febrile response 2–3 days after exposure leading to lethal viral encephalitis with animals reaching euthanasia endpoints at approximately 6 days after exposure. A pronounced granulocytosis was seen in the blood that corresponded with severe disease outcome. Fever, viremia, increases in granulocytes, and time to death were all similar to what we had previously reported with the cDNA clone-derived FL91-4679 strain, a mosquito isolate [14]. At lower doses of V105, macaques survived with little to no fever response.

EEG and ICP had not been previously measured during alphavirus infection of cynomolgus macaques, yet both methods can be used in the human clinical setting [7,9]. To determine if either physiological measurement provides an indicator of lethal EEEV disease, we developed a surgical method to implant radiotelemetry devices into macaques for infectious disease studies [16]. Significant changes in both EEG and ICP were only found in the lethally-infected macaques. There was a significant increase in the delta band that occurred simultaneously with the febrile period. This is similar to reports of an increase in slow-wave EEG activity during febrile encephalitis with EEEV in human patients [9,12]. The duration of febrile disease appeared to coincide with the suppression of circadian indices by EEG. Considerable time is required to extract and analyze EEG power bands from the telemetry data. Thus, EEG data is not useful in real-time as a biomarker for initiation of treatment or as an early indicator of outcome. However, in post-study data analysis and interpretation, EEG could be useful in evaluating the potential for vaccines or treatments to prevent or ameliorate CNS disease at otherwise lethal doses of EEEV.

ICP began to rise at the peak of the febrile response in lethally-infected macaques and continued to rise until the macaque was euthanized. Prior studies have reported an increased heart rate coupled with the febrile response and a late increase in blood pressure in severely diseased macaques [14, 29]. At this time, it is not clear if the rise in ICP is a result of the increase in blood pressure, swelling of the brain resulting from the encephalitis or increased cerebrospinal fluid in the CNS. Considering that the increase in ICP occurs well after fever onset, ICP may be more useful as a marker of outcome than as a potential trigger for initiation of treatment.

One potential biomarker for onset of lethal EEEV encephalitis was the day 4 rise in total WBC and granulocytes. Our 2007 report on the lethality of aerosolized EEEV in macaques also noted a rise in WBC and granulocytes that predicted outcome [14]. This rise occurs with the onset of the febrile response, therefore the rise in granulocytes could potentially be used in conjunction with fever as a ‘trigger’ for initiation of treatment in therapeutic efficacy studies. However, the therapeutic would have to be very effective, since by that point the virus has presumably entered the central nervous system and is replicating rapidly. Yet, CBC analysis may ultimately be a useful measure of systemic disturbance of homeostasis that, combined with amelioration of febrile responses, may indicate a broad impact of therapeutics or vaccines.

Extremely elevated levels of MCP-1, IP-10, and IL-8 were found in the brains of lethally-infected animals and likely contribute to the infiltration by peripheral leukocytes that were found at end-stage disease, including lymphocytes, NK cells, and macrophages. Remarkably, in surviving animals, the lymphocytes persist in the brain for at least 4 weeks after infection and recovery with no clinical signs of disease. This is the first study to document the phenotype of leukocytes migrating to the brain during EEEV encephalitis. Critical to further interpretation of this model will be serial euthanasia experiments where animals are euthanized at matched earlier time points (i.e. 6 dpi) for direct comparison of cytokines and cell infiltration into the CNS between animals receiving lethal or sublethal doses.

With regards to a potential biomarker detectable in the periphery, only IP-10 was elevated in the blood of lethal infections at 2 dpi, and levels were increased in only one of three lethally infected macaques. In mice infected with EEEV, IP-10 and other inflammatory cytokine/chemokine levels were only mildly elevated in the blood at 24 hours post-infection with wild-type virus [30], which was attributed to the binding of EEEV to heparan sulfate (41) and microRNA-mediated restriction or virus replication [31], both of which led to restricted infection of myeloid cells. The increased levels of IP-10 on day 2 post-infection may suggest further evaluation as a biomarker. Surprisingly, neither MCP-1 nor IL-8 levels were elevated in the blood despite the high levels found in the brain at necropsy and the pronounced granulocytosis noted in the blood and leukocyte infiltration into the brain. These changes of inflammatory cytokines and chemokines in the blood needs further evaluation before they would be considered suitable as a biomarker.

Closer examination of CNS tissues after necropsy also led to new findings that are of interest in understanding the pathophysiology of EEEV encephalitis. Vasculitis, which was reported in all of the CNS tissues in this study, was not a prominent finding in the original report of lethal aerosolized EEEV in cynomolgus macaques [14]. We also found high levels of inflammatory cytokines and chemokines in terminal samples from the brains of lethally-infected macaques by both multiplex and PCR. Lack of a similar response in plasma suggests that these host responses are generated within the brain parenchyma in response to the widespread virus infection of CNS cells. Furthermore, this response and the infiltration of leukocytes into the CNS may suggest that the immune response is contributing to the pathology in lethal EEEV infections. The fact that a robust cytokine/chemokine response is not observed in peripheral blood samples is consistent with the finding that EEEV infection is highly restricted in myeloid cells (in contrast with VEEV, for example) [30,31] although granulocytosis was observed. Perhaps most notable was the finding of viral RNA in the brains of four out of the nine surviving macaques, along with residual leukocytes. In fact, these four animals registered the highest number of fever-hours of all surviving animals. This finding would suggest that even in non-lethal infections of macaques, EEEV does reach the CNS and an inflammatory response is generated and may persist. The contribution of such phenomena to long term sequelae of EEEV infection, potentially even in unapparent cases, warrants intensive study.

In summary, we report here that an infectious clone of a human EEEV isolate, V105, is capable of inducing lethal viral encephalitis after aerosol exposure of macaques. EEG and ICP data collected by radiotelemetry in addition to body temperature would be useful in evaluating efficacy of medical countermeasures against EEEV in post-study data analysis. Because of how the EEG and ICP data are collected and the analysis required, at this time it would not be suitable for use as a trigger to initiate treatment with a potential therapeutic in an efficacy study. The combination of fever and granulocyte counts might be useful in conjunction as a treatment trigger. However, a comprehensive, multi-system measurement of vaccine or therapeutic efficacy could encompass all of the metrics explained above. Finally, we found evidence of overwhelming virus infection of the CNS in fatal EEEV coupled with a strong CNS inflammatory response, which will guide future research into the pathophysiology of EEEV.

## Materials and methods

### Ethics

The animal work performed adhered to the highest level of human animal care standards. The University of Pittsburgh is fully accredited by the Association for Assessment and Accreditation of Laboratory Animal Care (AAALAC). All animal work was performed under the standards of the Guide for the Care and Use of Laboratory Animals published by the National Institutes of Health and according to the Animal Welfare Act guidelines. All animal studies adhered to the principles stated in the Public Health Services Policy on Humane Care and Use of Laboratory Animals. The University of Pittsburgh Institutional Animal Care and Use Committee (IACUC) and the Department of the Army’s Animal Care and Use Review Office (U.S. Army Medical Research and Materiel Command [USAMRMC]) approved and oversaw the animal protocols for these studies (16026773, 1710064).

### Biological safety

All work with EEEV was conducted in the University of Pittsburgh Center for Vaccine Research (CVR) and the Regional Biocontainment Lab (RBL), which is currently registered and approved for work with EEEV by the Federal Select Agent Program (entity registration #C20110927-1269). All personnel have undergone appropriate Security Risk Assessment (SRA) and are approved through the University of Pittsburgh suitability assessment program. Respiratory protection for all personnel when handling infectious samples or working with animals is provided by powered air-purifying respirators (PAPRs; Versaflo TR-300; 3M, St. Paul, MN). Aerosol exposures were conducted within a class III biological safety cabinet (BSC). Liquid and surface disinfection was performed using Vesphene IIse detergent (dilution, 1:128; Steris Corporation, Erie, PA), while solid wastes, caging, and animal wastes were steam sterilized in an autoclave.

### Challenge virus

Macaques were infected via aerosol with EEEV alphavirus strain V105, derived from a cDNA clone based on GenBank accession number KP282670. V105 was originally isolated in 2005 from the brain of a fatal human case during an outbreak in Massachusetts [32, 33]. Viruses were produced initially by full-length, capped genome synthesis from linearized cDNA clones (mMessage Machine, Thermofisher) and electroporation of 10–20 μg of RNA into BHK 21 cells. Supernatants were harvested at 18–24 hours after electroporation and used to infect 1900 cm^2^ roller bottle cultures of Vero cells. At 18–24 hours after infection, Vero cell supernatants were harvested and subjected to low speed clarification prior to loading onto a 20%/60% discontinuous sucrose gradient in TNE buffer (10 mM Tris, 10 mM EDTA, 2 M NaCl, pH 7.4) and centrifugation for 3.5 hours at 24,000 RPM. The virus-containing interface was then harvested and virus particles were pelleted over a 20% sucrose (in TNE) gradient for 18–24 hours at 24,000 rpm. Pelleted virus was resuspended in Opti-MEM (Gibco 31985–070) and stored at -80 °C. Individual lots of virus were tested for virulence by aerosol dose-step infection of CD-1 mice and approved for NHP use only after typical virulence in mice (EEEV V105 LD_50_ ~200 PFU) was verified.

### General animal procedures

Cynomolgus macaques ranged from 2–9 years of age and included both male and female animals. Prior to use, the macaques were verified to be serologically negative for alphaviruses as well as Herpes B Virus, SIV, STLV, and SRV. Macaques were implanted with telemetry devices as described below. As needed, macaques were sedated for phlebotomy with 10mg/kg ketamine administered via intramuscular injection using a safety needle, and blood was collected from the femoral or saphenous vein. For euthanasia, macaques were sedated with 20 mg/kg ketamine followed by injection of sodium nitroprusside mixed with 12 ml of NaCl, followed by 200 mg/kg of Beuthanasia IV. Once euthanasia was confirmed, macaques were perfused via the left ventricle with saline.

### Telemetry implant surgery

Each macaque was equipped with a PhysioTel Digital radiotelemetry implant (Data Sciences International, DSI, St. Paul, MN) capable of continuously transmitting EEG, ICP, and temperature. Prior to implantation surgery, each macaque was anesthetized by injection of ketamine hydrochloride (20 mg/kg) and atropine (0.4 mg/kg); once anesthesia was confirmed they were maintained on ~1.5% isoflurane gas anesthesia for the duration of surgery. Each macaque received an intravenous (IV) catheter in the greater saphenous vein for 3% normal saline, a tracheal tube for intubation, with continuous pulse oximetry and rectal thermometry for vital sign monitoring. Prior to draping and fixture into a stereotaxic apparatus, the head, neck and upper back of each macaque was shaved and scrubbed in triplicate with betadine and chlorhexidine. Surgical implantation of the implant was performed as previously described [16]. Post-surgery, macaques were given analgesia and observed until recovered. At least 2 weeks after surgery macaques were transferred to the ABSL-3 facility.

### Aerosol exposure

Aerosol exposures were performed under the control of the Aero3G aerosol management platform (Biaera Technologies, Hagerstown, MD) as previously described [34]. Macaques were anesthetized with 6 mg/kg Telazol (Tiletamine HCl / Zolazepam HCl); once anesthesia was confirmed the macaque was weighed, bled, and transported to the Aerobiology suite using a mobile transport cart. The macaque was then transferred from the cart into a class III biological safety cabinet and the macaque’s head was placed inside a head-only exposure chamber. Jacketed External Telemetry Respiratory Inductive Plethysmography (JET-RIP; DSI) belts were placed around the upper abdomen and chest of the macaque and calibrated to a pneumotach. This allowed monitoring and recording of respiratory function during the exposure via the Ponemah software platform (DSI) during the aerosol [35]. Exposures were either 10 minutes in duration or accumulated tidal volume-based using the JET-RIP interface with the Biaera software. Aerosols were generated using an Aerogen Solo vibrating mesh nebulizer (Aerogen, Chicago, IL) as previously described, with a total airflow of 16.5 lpm of air into the chamber [36]. Total exhaust from the chamber including sampling was equal to intake air. To determine inhaled dose, an all-glass impinger (AGI; Ace Glass, Vineland, NJ) was attached to the chamber at operated at 6 lpm, -6 to -15 psi. Particle size was measured once during each exposure at 5 minutes using an Aerodynamic Particle Sizer (TSI, Shoreview, MN). A 5-minute air wash followed each aerosol before the macaque was removed from the cabinet, and transported back to its cage and observed until fully recovered from anesthesia. Virus concentration in nebulizer and AGI samples was assessed by plaque assay; inhaled dose was calculated as the product of aerosol concentration of the virus and the accumulated volume of inhaled air [37].

### EEG/ICP/Temperature data acquisition

Signals from DSI implants were transmitted to TRX-1 receivers connective via a communication link controller (CLC) to a computer running Ponemah v 5.2 or 5.3 (DSI). Closed circuit cameras (Axis Model No. M-1145) were positioned to continuously record macaque behavior including neurological abnormalities such as seizures and sleep disruption. Camera video was recorded through a Ponemah interface with MediaRecorder software (Noldus Information Technology, Leesburg, VA). Telemetry and video data was collected continuously from a baseline period (at least two days preceding aerosol challenge) until necropsy. At least once daily, data acquisition was stopped, data was transferred to a network server, and then acquisition was restarted.

### Temperature data analysis

Temperature data collected via radiotelemetry was exported from Ponemah as 15-minute averages into an Excel spreadsheet. Data was checked for missing or erroneous data points (body temperature <27oC or >43oC) and analyzed using an ARIMA model in MATLAB R2019A (MathWorks, Natick, MA). Prior to challenge, for each macaque the baseline temperature data was used to generate hourly temperature ranges for use in clinical scoring to determine significant deviations in temperature. Because the EEG/ICP implants were subcutaneous on the upper back of the macaque, temperature readings were lower than would be expected for core body temperatures but still exhibited 1–2°C diurnal variation prior to challenge. The code used is available on GitHub at https://github.com/ReedLabatPitt/Reed-Lab-Code-Library.

### EEG data analysis

Analysis of EEG and ICP data was performed as previously described [16]. Briefly, Ponemah data was opened in NeuroScore (v.3.0.0, DSI) software package, which facilitated batch processing of data to European Data Format (.edf) for analysis. EEG data was analyzed using in-house MATLAB (Mathworks, 2018a) scripts building on routines from the EEGLAB MATLAB toolbox EEGLAB [38]. Four distinct frequency bands were defined: delta [0.5 – 4Hz]; theta [4 – 8Hz]; alpha [8-12Hz]; and beta [12-30Hz] and estimated power in each band as the average power of all frequencies in the corresponding range. The EEG power spectral density magnitudes for each macaque were converted to percent change from baseline values across the whole of the specified periods: baseline, pre-symptomatic (0–2 dpi, inclusive), febrile (3–6 dpi, inclusive), and recovery (6+ dpi). Group averages of percent change at discrete frequencies were taken for severe and non-severe disease cohorts of macaques and compared against baseline by repeated measures ANOVA.

### Circadian index construction

To visualize changes in the circadian patterning of the time-resolved EEG power bands, a circadian index was constructed by subtracting the normalized beta power band from the normalized delta power band. Analysis of the periodicity of the circadian index was hampered by segments of missing data; to overcome this limitation, the Lomb-Scargle methodology of least-squares frequency analysis was employed to account for the gaps [39]. Circadian indices were compared via repeated measures ANOVA of their fundamental frequencies, or the lowest frequency of the associated waveform.

### ICP data analysis

ICP data was exported from Ponemah into MATLAB as time series data and grouped into mean daily averages to minimize noise; measurements were converted into percentage change from baseline, and repeated measures ANOVA was used to assess results for statistical significance.

### Clinical scoring

Macaques were scored twice daily by direct observation, at least six hours apart. Observations were increased for macaques that developed severe disease. The approved clinical scoring system consisted of three scoring categories. *Neurological score*: 1 = normal, 2 = occasional loss of balance or muscle control (ex: stumbling, unsteadiness), 3 = occasional nystagmus (eye oscillation/twitching), head pressing, tremors, 4 = loss of balance, nystagmus, head pressing, tremors; occasional seizures, 5 = frequent seizures, 6 = comatose (prompt euthanasia). *Activity score*: 1 = normal: responds to observer entering the room, frequent eye contact and body language interactions (both positive or aggressive postures/expressions); 2 = less active: responds to observer approaching the cage; body language interactions less frequent or intense; 3 = sluggish: only responds when prodded or when observer rattles the cage, maintains hunched posture with back to observer; limited eye contact and body language interactions; glassy eyes, grimace or sad facial expression; 4 = upright but inactive; does not respond to observer rattling the cage, ignores all stimuli; 5 = recumbent/moribund (prompt euthanasia). *Temperature score*: 1 = normal = baseline to 1.5 degrees above baseline; 2 = mild fever = 1.6–3.0 degrees above baseline; 3 = moderate fever = 3.1 to 4.0 degrees above baseline; 4 = severe fever = >4.0 degrees above baseline or mild hypothermia = 0.1–2 degrees below baseline; 5 = moderate hypothermia = 2.1–5 degrees below baseline; 6 = severe hypothermia = >5 degrees below baseline (prompt euthanasia). The daily clinical score for each macaque was the sum of the scores for these 3 criteria. Based on this scoring system, a healthy macaque’s baseline score was 3. A total score of 10 (3+3+4 on the 3 categories above) warranted observations every eight hours, including overnight. Macaques that reach a cumulative score of 14 were promptly euthanized. Email alerts on the Ponemah software were also used to notify study personnel of fever and severe hypothermia.

### Whole blood processing

Animals were sedated for sampling after infection by administration of 10 mg/kg ketamine via intramuscular injection. Once sedated, 2–3 ml of blood was drawn from either the right or left femoral vein into an EDTA tube. Plasma was frozen for serologic, immunologic and virologic assays. CBC analysis was performed using the Abaxis HM5 hematology analyzer. Blood chemistry analysis was performed using the Comprehensive Diagnostic Panel rotor (Abaxis 500–0038) on an Abaxis VS2 chemistry analyzer.

### Brain cell isolation

After perfusion of macaques with saline, brains were removed, weighed and used for cell isolation as described [40]. For each brain, 1 gram pieces dissected from the frontal, temporal, occipital, parietal, cerebellar regions were covered with digestion buffer consisting of modified HBSS without calcium and magnesium, 10mg/ml DNase I (Sigma 10104159001), 20mg/ml of collagenase (Sigma C2674) and mechanically digested using a scalpel. The sample was then incubated at 37°C for 45 minutes on a continuous rocker. Every 15 minutes, the sample was mechanically triturated using a serological pipet. The resulting homogenate was then filtered through a 40 um cell strainer and washed twice with wash buffer, consisting of HBSS with 3% FBS and 10 mg/ml DNase I and centrifuged at 500 x g for 8 minutes at room temperature. The supernatant was removed and the remaining pellet was suspended in 80% stock isotonic Percoll (SIP) (Sigma GE17-0891-01) made in HBSS solution. The suspension was subsequently overlaid with 10 ml of 38% SIP, 10 ml 21% SIP, followed by 5 ml HBSS with 3% FBS and centrifuged at 480 x gravity for 35 minutes, without brake. The third interface was removed, washed twice with modified HBSS containing 3% FBS and then suspended in 1 ml FACS buffer. Cells were counted using a hemocytometer and placed on ice. Cells suspended in FACS buffer were placed in a V-bottom 96-well plate and centrifuged at 500 x gravity for 4 minutes at 4°C. An Fc block was performed by adding 2 ul of purified anti-CD32 (BD Biosciences) and 18 ul of FACS buffer per sample for 20 minutes on ice in the dark. Cells were then washed twice with 200 ul FACS buffer and stained for live/dead. Cells were washed with 200 ul of FACS buffer and then stained antibody mix for 30 minutes on ice in the dark. Samples that required intracellular stain were permeabilized and fixed using BD Cytofix/Cytoperm (BD554714) and then washed with FACS perm-buffer. The stained samples were then washed twice with 200 ul FACS buffer followed by fixation with 200 ul of 4% PFA. Samples were run on a BD LSRII and analyzed using FlowJo 10.5.0.

### Cytokine analysis

Cytokine analysis was performed on plasma, CSF, and brain samples using Biolegend LEGENDplex 13-plex kit (Cat. No. 740389). Lethal brain samples were diluted 1:2 in assay buffer, plasma was run at 1:4 dilution, and convalescent brain or uninfected animals as well as all cerebral spinal fluid were run undiluted. Samples were prepared following LEGENDplex protocol for v-bottom kit. Samples were then run on FACSAria flow cytometer immediately following staining. Results were then analyzed using LEGENDplex Data Analysis Software. Analytes measured were interleukin (IL)-6, IL-10, IP-10, IL-β, IL-12p40, IL-17A, IFN-β, IL-23, TNF-α, IFN-γ, GM-CSF, IL-8 and MCP-1. An ELISA assay was used to measure MMP9 levels in plasma (R&D Systems; DMP900).

### Plaque assay

Viremia was determined by standard alphavirus plaque assay on BHK cells at each blood sampling time [41]. Briefly, plasma samples were diluted serially in 10-fold increments into phosphate buffered saline (PBS) with calcium/magnesium additive and 1% donor calf serum and then used to infect monolayers of BHK cells for 1 hour at 37 °C. Monolayers were then overlaid with 0.5% immunodiffusion agarose (MP Bio 952012) in BHK cell growth medium and plates were incubated at 37 °C for 24–48 hours. Monolayers were stained after 24–36 hours with 0.3 mM neutral red (Acros Organics 229810250) in PBS with 1% donor calf serum and plaques were enumerated.

### Plaque reduction neutralization test

Samples were diluted to 1:20 initially and then serially 2-fold into PBS with calcium/magnesium additive and 1% donor calf serum. Approximately 100 Vero cell PFU of EEEV V105 was then added to each dilution and incubated for 30 minutes at 37C. Each dilution was used to infect duplicate wells of Vero cells for one hour at 37 °C. After infection, a 0.5% immunodiffusion agarose overlay (in Vero cell growth medium) was added to each well and then the plates were incubated overnight (18–24 hours) at 37 °C. After incubation, the plates were stained with neutral red dye and plaques enumerated. PRNT values were calculated as the inverse of the dilution at which 50% or 80% of the plaques were reduced. A positive control consisted of anti-EEEV mouse ascites antibody purchased from the ATCC and a negative control was untreated virus dilutions.

### Tissue/Sample extraction and processing

Whole brain sections (frontal, parietal, temporal, occipital, cerebellum, thalamus), cervical lymph nodes (CLN), serum, cerebrospinal fluid (CSF), and nasal swabs were collected for EEEV control macaques. For whole brain sections and CLN, 100mg of tissue was harvested, suspended in 900 μL Tri-Reagent (Invitrogen), then homogenized using Omni tissue homogenizer (Omni International). For liquid samples, 100 μL of the specimen was suspended in 900μL Tri-Reagent, then thoroughly mixed by inversion. After a 10-minute incubation to ensure virus inactivation, the samples were transferred to a fresh tube prior to removal from tbe BSL-3 facility. Subsequent storage at -80°C or RNA isolation, cDNA synthesis, and qPCR analyses occurred in a BSL-2 setting.

### RNA isolation

RNA isolation was performed on whole tissue homogenate samples using a modified Invitrogen PureLink Viral RNA/DNA kit protocol. In brief, 200 μL of chloroform was added to the inactivated tissue homogenate/Tri-Reagent mixture, inverted vigorously for 30 seconds then centrifuged at 4°C at 12,000 RCF for 15 minutes to separate the organic phase from the RNA-containing aqueous phase. The aqueous phase was collected, mixed with an equal volume of 70% ethanol then applied to the PureLink spin column. For the remainder of the RNA isolation procedure, the Purelink Viral RNA/DNA kit protocol was used, including deoxyribonuclease treatment. RNA was eluted in RNase-free water to a final volume of 40 μL then stored at -80°C until further processing into cDNA.

For serum, CSF, and nasal swab samples, 5 μL of polyacryl carrier (Molecular Research Center) was added to the specimen/Tri-Reagent mixture, and incubated at room temperature for 30 seconds. Subsequently, 200 μL of chloroform was added to the mixture and incubated at room temperature for an additional 3 minutes, then centrifuged at 4°C at 12,000 RCF for 15 minutes. The aqueous phase was collected and combined with an equal volume of 100% isopropanol. The mixture was incubated at room temperature for 10 minutes before centrifugation at 4°C at 12,000 RCF for 10 minutes. The supernatant was removed and 1mL of 70% ethanol was added before further centrifugation at room temperature at 12,000 RCF to pellet the RNA. The supernatant was removed, and the pelleted RNA was resuspended in nuclease-free water. RNA was eluted in RNase-free water to a final volume of 40μL then stored at -80°C until further processing into cDNA.

### Complementary DNA (cDNA) synthesis

cDNA synthesis was performed using the First-strand cDNA Synthesis M-MLV Reverse Transcriptase Kit (Invitrogen) protocol, including RNaseOUT (Invitrogen). 5 μL of whole tissue samples (20 ng/μL) or undiluted plasma, CSF, or nasal swab RNA was added to the cDNA synthesis reaction. For quantification of EEEV vRNA, the following cDNA synthesis primers with a T7 promoter tag (bolded) targeting the positive strand was used: EEEV (V105) 5’- GCGTAATACGACTCACTATACACCGGCCAAAGTCTTCCATACTAT-3’ (IDT). Primers targeted the EEEV NSP2 coding region. For quantification of cytokines, chemokines, or proteases, random oligonucleotide primers (ThermoFisher) were used to reverse transcribe all RNA using the First-strand cDNA Synthesis M-MLV Reverse Transcriptase Kit protocol. Thermocycler parameters recommended by the manufacturer were utilized. cDNA was stored at -80°C until further analysis via qPCR.

### qRT-PCR for viral genomes

For quantitation of genomic vRNA, qRT-PCR was performed using the 2x Fast TaqMan Universal PCR Master Mix, No AmpErase UNG (Applied Biosystems), following the manufacturer’s instructions. Forward primer and probe targeting EEEV include: forward primer 5’-GCGCTACAAGGTCAATGAGA-3’ and probe 5’-ACGCACAGACATCTGAGCATGTGAA-3’. The probe was labeled at the 5’ end with the reporter molecule 6-carboxyfluorescein (6-FAM) and quenched internally at a modified thymidine residue with Black Hole Quencher (BHQ1), with a modified 3’ end to prevent probe extension by Taq polymerase. Reverse primer for EEEV targeted the T7 promoter tag introduced during cDNA synthesis: 5’-GCGTAATACGACTCACTATA-3’. Thermocycler parameters consist of initial denaturing: 95°C for 20 seconds, and cycling PCR amplification (45 cycles): 95°C for 3 seconds and 60°C for 20 seconds. Quantitation of virus was determined by comparing the cycle threshold (CT) values from unknown samples to CT values from positive-sense EEEV vRNA standard curve from 1:10 serial dilutions. Positive-sense vRNA was developed in-house by in vitro transcription, using the mMessage mMachine T7 kit (Ambion) and following the manufacturer’s instructions [42]. Limit of detection (LOD) determination was carried out in accordance with the Minimum Information for the Publication of Quantitative Real-Time PCR Experiments (MIQE) guidelines [43]. The LOD for EEEV qPCR was 760 genome copies and the Final LODs based on 1 mL or 1 mg of sample is 6.08 x 10^4^ genome copies/mL or mg.

### Semi-quantitative RT-PCR

2x Fast Taqman Universal PCR Master Mix, No AmpErase UNG and Taqman Gene Expression Assay kits (Invitrogen) were used for semi-quantitative analysis of cytokines, chemokines and proteases (MMP-9, IL-1β, MCP-1, IP-10, IFN-γ. IL-6, IL-8) within whole brain tissues, following manufacturer’s instructions. Probes were labeled at the 5’ end with t 6-FAM and quenched with BHQ1, with a modified 3’ end to prevent probe extension by Taq polymerase. Thermocycling parameters comprised the following: hold step, 50°C for 120 seconds; initial denaturing, 95°C for 120 seconds; and cycling PCR amplification, 95°C for 1 second and 60°C for 20 seconds (40 cycles). All kits were targeted to cynomolgus macaque-specific, highly-conserved amplicons that spanned one or more exons to guarantee desired mRNA transcript specificity. Endogenous controls comprised TaqMan Gene Expression Assay kits for GAPDH and β-actin, with corresponding brain tissue from a mock-infected macaque serving as the reference tissue. Inter-run calibration to normalize inter-run/inter-plate variability was carried out according to best practices [43, 44].

### Traumatic brain injury gene expression measurements

TaqMan Gene Expression Assay kits (ThermoFisher) were used to target genes of interest (GFAP Rh00909240_m1; MMP9 Mf02856273_g1; LIF Mf04365516_m1). Probes were labeled at the 5’ end with the reporter molecule 6-carbocyfluorescein (6-FAM) and quenched internally at a modified “T” residue with BHQ1 (Black Hole Quencher), with a modified 3’ end to prevent probe extension by Taq polymerase. Thermocycling parameters comprised the following: hold step, 50°C for 120 seconds; initial denaturing, 95°C for 120 seconds; and cycling PCR amplification, 95°C for 1 second and 60°C for 20 seconds (40 cycles). Endogenous controls comprised TaqMan Gene Expression Assay kits for GAPDH, with thalamus tissue from a mock-infected macaque serving as the reference tissue; endogenous controls for genes of circadian regulation employed 18S rRNA as a time-invariant housekeeping gene. Inter-run calibration to normalize inter-run/inter-plate variability was carried out according to best practices [43, 44].

### Pathology

Tissue samples were fixed with 10% neutral buffered formalin according to approved inactivation protocols. Upon removal from BSL-3, samples were embedded in paraffin blocks and sectioned onto slides. Slides were stained with hematoxylin and eosin, followed by pathology scoring. Slides were assessed for leukocyte infiltration (including meningitis and vasculitis), hemorrhage, and loss of neurons. Lesions were scored as 0 = normal, 1 = mild, 2 = moderate, 3 = severe, 4 = widespread and severe.

### Statistics

Graphpad Prism was used for statistical determinations. SPSS was used for LD_50_ determination. Graphpad Prism and MATLAB were used to analyze the EEG and ICP data sets for statistical significance.

## Supporting information

S1 Fig Flow gating strategy for brain leukocyte phenotyping. Both panels started with viable cells and singlet inclusion. In the lymphoid panel, CD45+ cells were further segregated into T cells using CD3, CD4, and CD8. B cells were identified by CD20+HLA-DR+. NK cells were identified by NKG2A expression. In the myeloid panel, CD45+ cells were identified by CD14 and CD16 expression. Classical monocytes were CD14+CD16-, intermediate monocytes were CD14+CD16+, and non-classical were CD14-CD16+. Classical monocytes were further subdivided based on CCR2 and Iba-1 expression. Infiltrating macrophages were CD14+CD16-CD11b+HLA-DR+Iba-1-CCR2+. Resident microglia were identified by CD14+CD16-CD11b+HLA-DR+Iba-1+CCR2-. Microglia-like macrophages were identified by CD14+CD16-CD11b+HLA-DR+Iba-1+CCR2+.
(TIF)

## References

[1] Zacks MA, Paessler S. Encephalitic alphaviruses. Vet Microbiol. 2010;140(3–4):281–6. Epub 2009/09/25. 10.1016/j.vetmic.2009.08.023 . 19775836 PMC2814892

[2] Young DS, Kramer LD, Maffei JG, Dusek RJ, Backenson PB, Mores CN, et al Molecular epidemiology of eastern equine encephalitis virus, New York. Emerg Infect Dis. 2008;14(3):454–60. Epub 2008/03/08. 10.3201/eid1403.070816 . 18325261 PMC2570827

[3] Hollidge BS, Gonzalez-Scarano F, Soldan SS. Arboviral encephalitides: transmission, emergence, and pathogenesis. J Neuroimmune Pharmacol. 2010;5(3):428–42. Epub 2010/07/24. 10.1007/s11481-010-9234-7 . 20652430 PMC3286874

[4] Morens DM, Folkers GK, Fauci AS. Eastern Equine Encephalitis Virus—Another Emergent Arbovirus in the United States. New England Journal of Medicine. 2019;381(21):1989–92. 10.1056/NEJMp1914328 31747726

[5] Goldfield M, Sussman O. The 1959 outbreak of Eastern encephalitis in New Jersey. I. Introduction and description of outbreak. Am J Epidemiol. 1968;87(1):1–10. Epub 1968/01/01. 10.1093/oxfordjournals.aje.a120789 . 5637871

[6] Clarke DH. Two nonfatal human infections with the virus of eastern encephalitis. Am J Trop Med Hyg. 1961;10:67–70. Epub 1961/01/01. 10.4269/ajtmh.1961.10.67 . 13693885

[7] Wendell LC, Potter NS, Roth JL, Salloway SP, Thompson BB. Successful management of severe neuroinvasive eastern equine encephalitis. Neurocrit Care. 2013;19(1):111–5. Epub 2013/06/05. 10.1007/s12028-013-9822-5 . 23733173

[8] De Webster HF. Eastern equine encephalomyelitis in Massachusetts; report of two cases, diagnosed serologically, with complete clinical recovery. N Engl J Med. 1956;255(6):267–70. Epub 1956/08/09. 10.1056/NEJM195608092550604 . 13348851

[9] Silverman MA, Misasi J, Smole S, Feldman HA, Cohen AB, Santagata S, et al Eastern equine encephalitis in children, Massachusetts and New Hampshire,USA, 1970–2010. Emerg Infect Dis. 2013;19(2):194–201; quiz 352. Epub 2013/01/25. 10.3201/eid1902.120039 . 23343480 PMC3559032

[10] Reddy AJ, Woods CW, Welty-Wolf KE. Eastern equine encephalitis leading to multi-organ failure and sepsis. J Clin Virol. 2008;42(4):418–21. Epub 2008/05/06. 10.1016/j.jcv.2008.03.008 . 18456547

[11] Przelomski MM, O’Rourke E, Grady GF, Berardi VP, Markley HG. Eastern equine encephalitis in Massachusetts. A report of 16 cases, 1970–1984. 1988;38(5):736-. 10.1212/wnl.38.5.736 3362371

[12] Deresiewicz RL, Thaler SJ, Hsu L, Zamani AA. Clinical and neuroradiographic manifestations of eastern equine encephalitis. N Engl J Med. 1997;336(26):1867–74. Epub 1997/06/26. 10.1056/NEJM199706263362604 . 9197215

[13] Wyckoff RWG, Tesar WC. Equine Encephalomyelitis in Monkeys. The Journal of Immunology. 1939;37(4):329.

[14] Reed DS, Lackemeyer MG, Garza NL, Norris S, Gamble S, Sullivan LJ, et al Severe encephalitis in cynomolgus macaques exposed to aerosolized Eastern equine encephalitis virus. J Infect Dis. 2007;196(3):441–50. Epub 2007/06/29. 10.1086/519391 . 17597459

[15] Food and Drug Administration (FDA). 21 CFR Parts 314 and 601. Evidence Needed to Demonstrate Effectiveness of New Drugs When Human Efficacy Studies are Not Ethical or Feasible; final rule. Federal Register. 2002;67(105):37988–98. 12049094

[16] Ma H, Lundy JD, Cottle EL, O’Malley KJ, Trichel AM, Klimstra WB, et al Applications of minimally invasive multimodal telemetry for continuous monitoring of brain function and intracranial pressure in macaques with acute viral encephalitis. PLOS ONE. 2020;15(6):e0232381 10.1371/journal.pone.0232381 32584818 PMC7316240

[17] Ludlow M, Kortekaas J, Herden C, Hoffmann B, Tappe D, Trebst C, et al Neurotropic virus infections as the cause of immediate and delayed neuropathology. Acta Neuropathologica. 2016;131(2):159–84. 10.1007/s00401-015-1511-3 26659576 PMC4713712

[18] Kennedy PG. Viral encephalitis: causes, differential diagnosis, and management. J Neurol Neurosurg Psychiatry. 2004;75 Suppl 1(Suppl 1):i10–5. Epub 2004/02/24. 10.1136/jnnp.2003.034280 . 14978145 PMC1765650

[19] Ferri R, Cosentino FI, Elia M, Musumeci SA, Marinig R, Bergonzi P. Relationship between Delta, Sigma, Beta, and Gamma EEG bands at REM sleep onset and REM sleep end. Clin Neurophysiol. 2001;112(11):2046–52. Epub 2001/10/30. 10.1016/s1388-2457(01)00656-3 . 11682342

[20] Rachalski A, Authier S, Bassett L, Pouliot M, Tremblay G, Mongrain V. Sleep electroencephalographic characteristics of the Cynomolgus monkey measured by telemetry. J Sleep Res. 2014;23(6):619–27. Epub 2014/08/12. 10.1111/jsr.12189 . 25109588

[21] Authier S, Paquette D, Gauvin D, Sammut V, Fournier S, Chaurand F, et al Video-electroencephalography in conscious non human primate using radiotelemetry and computerized analysis: refinement of a safety pharmacology model. J Pharmacol Toxicol Methods. 2009;60(1):88–93. Epub 2009/05/06. 10.1016/j.vascn.2008.12.003 . 19414069

[22] Gwin JT, Ferris DP. Beta- and gamma-range human lower limb corticomuscular coherence. Frontiers in human neuroscience. 2012;6:258-. 10.3389/fnhum.2012.00258 . 22973219 PMC3438504

[23] Keren AS, Yuval-Greenberg S, Deouell LY. Saccadic spike potentials in gamma-band EEG: characterization, detection and suppression. Neuroimage. 2010;49(3):2248–63. Epub 2009/10/31. 10.1016/j.neuroimage.2009.10.057 . 19874901

[24] Krause G, Ullsperger P, Beyer L, Gille HG. Changes in EEG power density spectrum during static muscle work. European Journal of Applied Physiology and Occupational Physiology. 1983;51(1):61–6. 10.1007/BF00952538 6684033

[25] Bauer S, Rasika S, Han J, Mauduit C, Raccurt M, Morel G, et al Leukemia inhibitory factor is a key signal for injury-induced neurogenesis in the adult mouse olfactory epithelium. J Neurosci. 2003;23(5):1792–803. Epub 2003/03/12. 10.1523/JNEUROSCI.23-05-01792.2003 . 12629183 PMC6741956

[26] Higashida T, Kreipke CW, Rafols JA, Peng C, Schafer S, Schafer P, et al The role of hypoxia-inducible factor-1α, aquaporin-4, and matrix metalloproteinase-9 in blood-brain barrier disruption and brain edema after traumatic brain injury. J Neurosurg. 2011;114(1):92–101. Epub 2010/07/14. 10.3171/2010.6.JNS10207 . 20617879

[27] Klapka N, Müller HW. Collagen matrix in spinal cord injury. J Neurotrauma. 2006;23(3–4):422–35. Epub 2006/04/25. 10.1089/neu.2006.23.422 . 16629627

[28] Manek R, Moghieb A, Yang Z, Kumar D, Kobessiy F, Sarkis GA, et al Protein Biomarkers and Neuroproteomics Characterization of Microvesicles/Exosomes from Human Cerebrospinal Fluid Following Traumatic Brain Injury. Mol Neurobiol. 2018;55(7):6112–28. Epub 2017/12/01. 10.1007/s12035-017-0821-y . 29188495 PMC6359938

[29] Ma H, Lundy JD, O’Malley KJ, Klimstra WB, Hartman AL, Reed DS. Electrocardiography Abnormalities in Macaques after Infection with Encephalitic Alphaviruses. Pathogens. 2019;8(4):240 10.3390/pathogens8040240 31744158 PMC6969904

[30] Gardner CL, Ebel GD, Ryman KD, Klimstra WB. Heparan sulfate binding by natural eastern equine encephalitis viruses promotes neurovirulence. Proc Natl Acad Sci U S A. 2011;108(38):16026–31. Epub 2011/09/08. 10.1073/pnas.1110617108 . 21896745 PMC3179095

[31] Trobaugh DW, Gardner CL, Sun C, Haddow AD, Wang E, Chapnik E, et al RNA viruses can hijack vertebrate microRNAs to suppress innate immunity. Nature. 2014;506(7487):245–8. Epub 2013/12/20. 10.1038/nature12869 . 24352241 PMC4349380

[32] Centers for Disease Control and Prevention (CDC). Eastern equine encephalitis—New Hampshire and Massachusetts, August-September 2005. MMWR Morb Mortal Wkly Rep. 2006;55(25):697–700. Epub 2006/07/01. . 16810146

[33] Yu G-Y, Wiley MR, Kugelman JR, Ladner JT, Beitzel BF, Eccleston LT, et al Complete coding sequences of eastern equine encephalitis virus and venezuelan equine encephalitis virus strains isolated from human cases. Genome Announc. 2015;3(2):e00243–15. 10.1128/genomeA.00243-15 . 25908124 PMC4408325

[34] Hartman AL, Powell DS, Bethel LM, Caroline AL, Schmid RJ, Oury T, et al Aerosolized Rift Valley Fever Virus Causes Fatal Encephalitis in African Green Monkeys and Common Marmosets. Journal of Virology. 2014;88(4):2235 10.1128/JVI.02341-13 24335307 PMC3911574

[35] Bohannon JK, Janosko K, Holbrook MR, Barr J, Pusl D, Bollinger L, et al Safety Precautions and Operating Procedures in an (A)BSL-4 Laboratory: 3. Aerobiology. J Vis Exp. 2016;(116). Epub 2016/10/22. 10.3791/53602 . 27768036 PMC5092082

[36] Bowling JD, O’Malley KJ, Klimstra WB, Hartman AL, Reed DS. A Vibrating Mesh Nebulizer as an Alternative to the Collison Three-Jet Nebulizer for Infectious Disease Aerobiology. Appl Environ Microbiol. 2019;85(17). Epub 2019/06/30. 10.1128/AEM.00747-19 . 31253680 PMC6696971

[37] Roy CJ, Reed DS. Infectious disease aerobiology: miasma incarnate. Front Cell Infect Microbiol. 2012;2:163 Epub 2012/12/26. 10.3389/fcimb.2012.00163 . 23267441 PMC3525905

[38] Delorme A, Makeig S. EEGLAB: an open source toolbox for analysis of single-trial EEG dynamics including independent component analysis. J Neurosci Methods. 2004;134(1):9–21. Epub 2004/04/23. 10.1016/j.jneumeth.2003.10.009 . 15102499

[39] Lomb NR. Least-squares frequency analysis of unequally spaced data. Astrophysics and Space Science. 1976;39(2):447–62. 10.1007/BF00648343

[40] Albe JR, Boyles DA, Walters AW, Kujawa MR, McMillen CM, Reed DS, et al Neutrophil and macrophage influx into the central nervous system are inflammatory components of lethal Rift Valley fever encephalitis in rats. PLoS Pathog. 2019;15(6):e1007833 Epub 2019/06/21. 10.1371/journal.ppat.1007833 . 31220182 PMC6605717

[41] Klimstra WB, Ryman KD, Johnston RE. Adaptation of Sindbis virus to BHK cells selects for use of heparan sulfate as an attachment receptor. J Virol. 1998;72(9):7357–66. Epub 1998/08/08. 10.1128/JVI.72.9.7357-7366.1998 . 9696832 PMC109960

[42] Gardner CL, Burke CW, Tesfay MZ, Glass PJ, Klimstra WB, Ryman KD. Eastern and Venezuelan equine encephalitis viruses differ in their ability to infect dendritic cells and macrophages: impact of altered cell tropism on pathogenesis. J Virol. 2008;82(21):10634–46. Epub 2008/09/05. 10.1128/JVI.01323-08 . 18768986 PMC2573165

[43] Bustin SA, Benes V, Garson JA, Hellemans J, Huggett J, Kubista M, et al The MIQE guidelines: minimum information for publication of quantitative real-time PCR experiments. Clin Chem. 2009;55(4):611–22. Epub 2009/02/28. 10.1373/clinchem.2008.112797 . 19246619

[44] Hellemans J, Mortier G, De Paepe A, Speleman F, Vandesompele J. qBase relative quantification framework and software for management and automated analysis of real-time quantitative PCR data. Genome Biol. 2007;8(2):R19 Epub 2007/02/13. 10.1186/gb-2007-8-2-r19 . 17291332 PMC1852402

