# Supplementary figures and images for: Physiological and immunological changes in the brain associated with lethal eastern equine encephalitis virus in macaques
Source: PLoS Pathog. 2021 Feb 3;17(2):e1009308. doi: 10.1371/journal.ppat.1009308 (PMC7886169; doi:10.1371/journal.ppat.1009308)

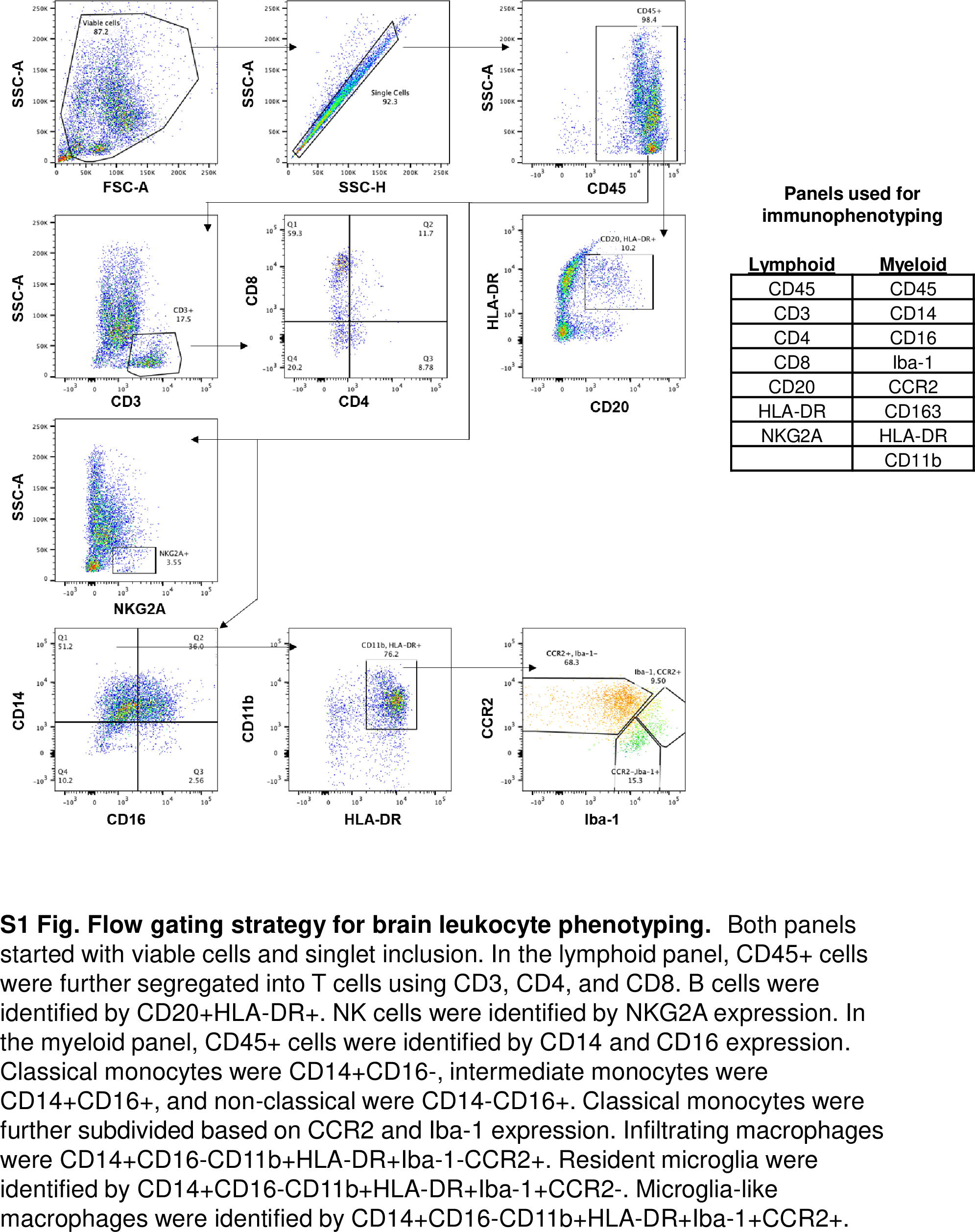

Supplement: S1 Fig — Both panels started with viable cells and singlet inclusion. In the lymphoid panel, CD45+ cells were further segregated into T cells using CD3, CD4, and CD8. B cells were identified by CD20+HLA-DR+. NK cells were identified by NKG2A expression. In the myeloid panel, CD45+ cells were identified by CD14 and CD16 expression. Classical monocytes were CD14+CD16-, intermediate monocytes were CD14+CD16+, and non-classical were CD14-CD16+. Classical monocytes were further subdivided based on CCR2 and Iba-1 expression. Infiltrating macrophages were CD14+CD16-CD11b+HLA-DR+Iba-1-CCR2+. Resident microglia were identified by CD14+CD16-CD11b+HLA-DR+Iba-1+CCR2-. Microglia-like macrophages were identified by CD14+CD16-CD11b+HLA-DR+Iba-1+CCR2+. (TIF) [file ppat.1009308.s001.tif]
